# The Yin and Yang of pathogens and probiotics: interplay between *Salmonella enterica* sv. Typhimurium and *Bifidobacterium infantis* during co-infection

**DOI:** 10.3389/fmicb.2024.1387498

**Published:** 2024-05-15

**Authors:** Claire Shaw, Bart C. Weimer, Reed Gann, Prerak T. Desai, Jigna D. Shah

**Affiliations:** Department of Population Health and Reproduction, School of Veterinary Medicine, 100K Pathogen Genome Project, University of California, Davis, Davis, CA, United States

**Keywords:** probiotic, arginine, nitric oxide, host-microbe association, *Salmonella*, bifidobacteria

## Abstract

Probiotic bacteria have been proposed as an alternative to antibiotics for the control of antimicrobial resistant enteric pathogens. The mechanistic details of this approach remain unclear, in part because pathogen reduction appears to be both strain and ecology dependent. Here we tested the ability of five probiotic strains, including some from common probiotic genera *Lactobacillus* and *Bifidobacterium*, to reduce binding of *Salmonella enterica* sv. Typhimurium to epithelial cells *in vitro. Bifidobacterium longum* subsp. *infantis* emerged as a promising strain; however, *S.* Typhimurium infection outcome in epithelial cells was dependent on inoculation order, with *B. infantis* unable to rescue host cells from preceding or concurrent infection. We further investigated the complex mechanisms underlying this interaction between *B. infantis*, *S.* Typhimurium, and epithelial cells using a multi-omics approach that included gene expression and altered metabolism via metabolomics. Incubation with *B. infantis* repressed apoptotic pathways and induced anti-inflammatory cascades in epithelial cells. In contrast, co-incubation with *B. infantis* increased in *S.* Typhimurium the expression of virulence factors, induced anaerobic metabolism, and repressed components of arginine metabolism as well as altering the metabolic profile. Concurrent application of the probiotic and pathogen notably generated metabolic profiles more similar to that of the probiotic alone than to the pathogen, indicating a central role for metabolism in modulating probiotic-pathogen-host interactions. Together these data imply crosstalk via small molecules between the epithelial cells, pathogen and probiotic that consistently demonstrated unique molecular mechanisms specific probiotic/pathogen the individual associations.

## Introduction

The gut microbiome provides a first line of defense against enteric infections. This essential community of microorganisms is also easily disrupted by dietary and local ecological factors that provide an opportunity for enteric pathogens to cause disease (Ducarmon et al., 2019). One proposed mechanism for the maintenance or reestablishment of a normal gut microbiome after community perturbations is the use of probiotic bacteria (Gareau et al., 2010). An important and accepted use of probiotics is to control enteric infections, which is especially timely given the rising number of multidrug resistant pathogens and subsequently dwindling number of effective antibiotics (Paton et al., 2006; Kulkarni et al., 2022). One notable candidate for probiotic modulation is multidrug resistant foodborne pathogen *Salmonella enterica* sv. Typhimurium, which causes over 80 million cases annual of foodborne illness globally and is a particular threat to infant mortality (Gong et al., 2022). Globally the burden of enteric pathogens is particularly prominent in the infant population, as evidenced by large cohort studies surveying disease incidence in children (Majowicz et al., 2010; Kotloff et al., 2013; Kasumba et al., 2021; Kotloff, 2022). In this high-risk infant population, currently a target population for probiotic development, *Salmonella*-induced diarrhea is a particularly notable issue with severe and potentially fatal health consequences if left untreated, especially in low-and middle-income countries (CDC, 2008; Majowicz et al., 2010; Shkalim et al., 2012; Kasumba et al., 2021; Eor et al., 2023; Splichalova et al., 2023). The need to find antibiotic alternatives for enteric diseases is urgent as antimicrobial resistance is increasing along with the evolution of multi-drug resistant strains (Nair et al., 2018; NARMS, 2023), with probiotics poised to be one such alternate treatment.

Though the mechanism by which probiotic organisms mitigate enteric infections is not yet well understood, current literature suggests probiotics may alleviate infection by acting as an immune stimulant, bioactive metabolite production, modulating gut pH, competing for nutrients used for infection, physically blocking host receptors, or through a combination of these activities (Lebeer et al., 2008; Iqbal et al., 2021). Supporting the potential immunomodulatory role of some probiotics, one study of *S.* Typhimurium infection in a mouse model indicated *Lacticaseibacillus casei* probiotic treatment not only decreased neutrophil infiltration and inflammation, but also increased the release of *S.* Typhimurium specific IgA in the intestinal lumen (de LeBlanc Ade et al., 2010). Other studies have more conflicting results that suggest probiotic efficacy and mechanistic underpinnings are context and host specific. Work on *B. longum* administration in one mouse model of *salmonellosis* revealed probiotic administration increased survival rate but had no impact of fecal shedding of the pathogen (Silva et al., 2004), while another study on the use of lactoferrin and milk-derived protein interventions displayed a reduction in *Salmonella* shedding and diarrhea in a porcine model (Hu et al., 2012). Together these results suggest that molecular signaling is part of the infection paradigm with unclear biological rules that may be dominated by strain variation represented by the large genomic variation among the bacterial actors (Arboleya et al., 2018; Díaz et al., 2021; van Puyvelde et al., 2023; Zhou et al., 2023). Though previous findings suggest probiotics are a possible treatment strategy for salmonellosis, they present their own set of challenges, such as differing adaptability and suitability to the target environment (Yu et al., 2013; Mercer and Arrieta, 2023; Thorman et al., 2023). To potentiate the best chance of success against enteric colonization, probiotics must be well-suited, even isolated from, their target environment. With this in mind, bifidobacteria stand as one probiotic option in *Salmonella*-susceptible population of infants (Mills et al., 2023).

Bifidobacteria are among the early colonizers of the infant gut, along with other commensal bacteria that include *Escherichia coli, Staphylococcus,* and *Streptococcus* (Mueller et al., 2015; Secher et al., 2016; Stewart et al., 2018). Around 40–60% of the fecal microbiota of a 2-week-old infant are *Bifidobacterium* species, though some formula-fed infants have no detectable bifidobacteria while other breast-fed infants show up to 90% bifidobacteria in their stool microbiome (di Gioia et al., 2014). This specificity of *Bifidobacterium* to the infant gut (LoCascio et al., 2010; Marcobal et al., 2011; Frese et al., 2017) coupled with the ability of some species to alleviate or prevent salmonellosis makes bifidobacteria a robust candidate as a potential infant probiotic. It is imperative that a mechanistic understanding be elucidated of how bifidobacteria may inhibit *Salmonella* considering the targeted infant population for such application are immune naïve. An important part of understanding the potential efficacy of probiotics like bifidobacteria is developing an improved understanding of the interaction between host, pathogen, and the gut microbiome at the host mucosa (Turroni et al., 2010; Pudlo et al., 2015; Underwood et al., 2015).

Many studies have sought to unravel the individual effect of probiotics, commensals, or pathogens on the host, but probiotic-pathogen interactions at the host interface remains an understudied field that is being complicated by the genomic variation of *Salmonella* (Gupta et al., 2019), strain variation in bifidobacteria (LoCascio et al., 2010; Díaz et al., 2021), and the variation in ecology of the gut between age groups (Voreades et al., 2014; Laforest-Lapointe and Arrieta, 2017). A steady increase in literature suggesting interspecies communication (i.e., community cross talk) exerts some control over the expression of virulence genes in enteric pathogens further highlights the need to define biochemical and genomic underpinnings of these interactions (Kendall and Sperandio, 2007; Shaw et al., 2022). The modulation of microbial activity by neighboring gut microbes via chemical signaling, for instance by widely conserved quorum response transcription factor *sdiA* in *Salmonella* (Plitnick et al., 2021), suggests microbe-microbe interactions via small metabolites are a significant factor in the infection process of enteric pathogens and potentially also in the response to probiotic interactions (Thompson et al., 2016).

Some probiotic bacteria have been suggested to potentiate positive host health effects via the production and regulation of bioactive metabolites (Indira et al., 2019). Metabolic regulation is also a determining factor in the success of *Salmonella* infection (Ilyas et al., 2017; Herrero-Fresno and Olsen, 2018). *Salmonella* is a metabolically flexible enteric pathogen known to manipulate host metabolism for pathogenic gain (Hume et al., 2017). Considering the known importance of metabolism in regulating *Salmonella* infection, the potential modulation of the host-microbe metabolic landscape by probiotic administration is a particularly intriguing mechanism to investigate.

Here we hypothesize that the addition of *B. infantis* to gut epithelial cells will affect the infection-related metabolic response of *S.* Typhimurium, attenuating adhesion and invasion of host cells. We subsequently show the relationship between *B. infantis*-*S.* Typhimurium was dependent on multiple factors, such as order of inoculation (concurrent addition, staggered addition, or co-incubation) that affect the ability of *B. infantis* to attenuate *S.* Typhimurium virulence. This study explored the complexity of these probiotic-pathogen interactions through evaluation of host, probiotic, and pathogen gene expression, and through small metabolite profiles, ultimately revealing the host-protective effects of *B. infantis* are both species and context dependent. Together the data suggest probiotic efficacy is reliant on the existence of narrow circumstances and thus must be more fully explored in lab settings, as demonstrated here, prior to clinical use.

## Results

### Probiotic strain selection

**Figure 1:**
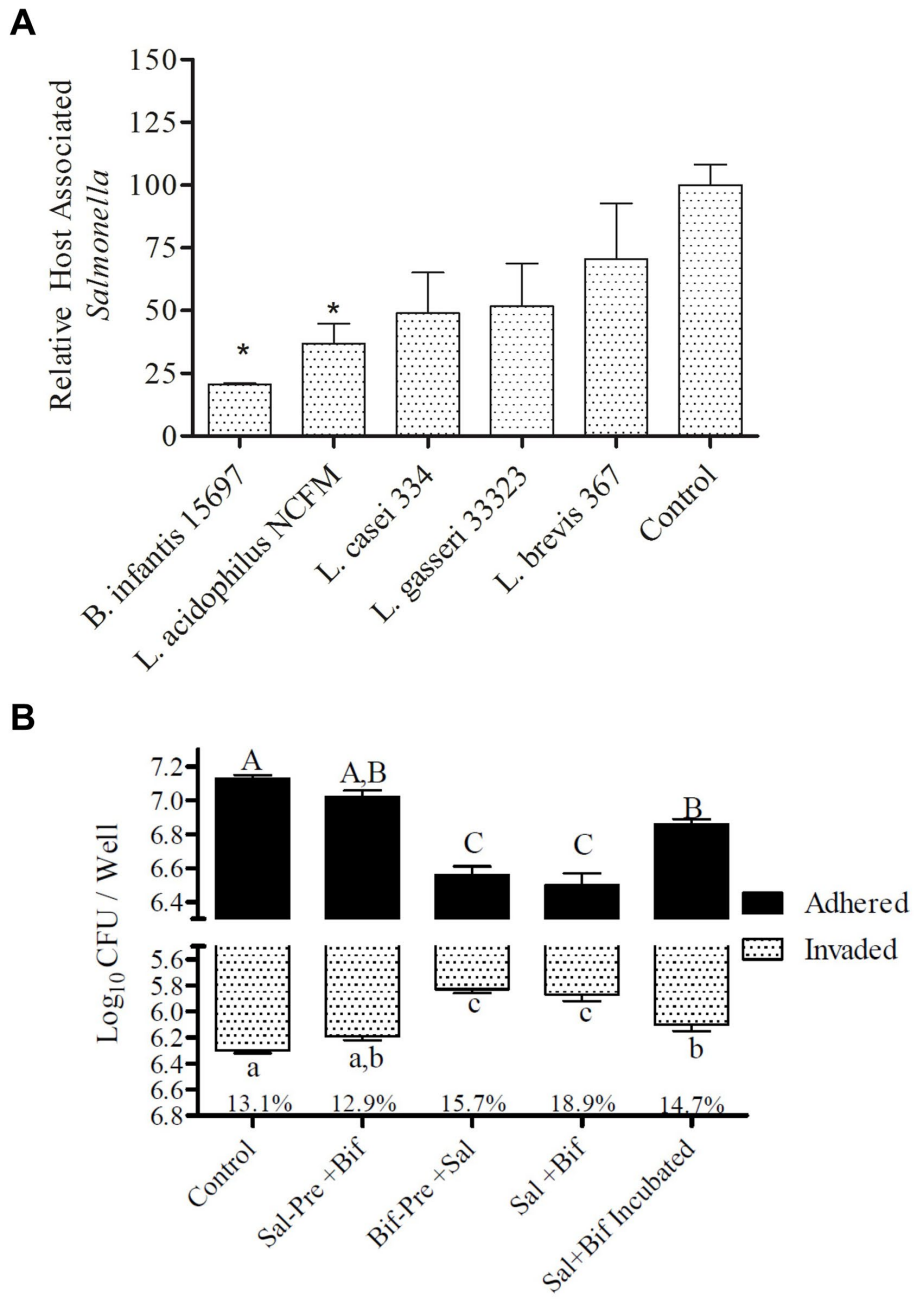
Adhesion to and invasion of Caco2 cells by *S.* Typhimurium. **(A)** Efficacy of different strains t block *Salmonella* host association. Treatments with “*” are significantly different (*p* ≤ 0.5) as compared to control (Caco2 cells incubated with *Salmonella alone*). *Error* bars indicate standard error from 4 replicates. **(B)** Effect of presence of *B. infantis* on the relative adhesion and invasion of *S.* Typhimurium to Caco2 cells. Control (Caco2 cells incubated with *S.* Typhimurium); Sal-Pre + Bif (Caco2 cells pre-incubated for 30 min with *S.* Typhimurium and then with *B. infantis* for 30 more minutes); Bif-Pre+ Sal (Caco2 cells pre-incubated with *B. infantis* for 30 minutes and then with *S.* Typhimurium for 60 more minutes); Sal+Bif (Caco2 cells incubated with *S.* Typhimurium and *B. infantis* together for 60 minutes); Sal+Bif Incubated (*S.* Typhimurium and *B. infantis* were first incubated together for 60 minutes and then incubated with Caco2 cells for 120 minutes). Treatments that do not share an alphabet are significantly different (p ≤ 0.05). The % numbers of the x axis represents the fraction of total host associated bacteria that were intracellular (invaded). Error bard indicate standard error from three replicates.

Five different probiotic organisms, *Bifidobacterium longum* spp. *infantis* (ATCC 15697)*, Lactobacillus acidophilus* (NCFM)*, Lactocaseibacillus casei* (ATCC 334)*, Lactobacillus gasseri* (ATCC 33323), and *Levilactobacillus brevis* (ATCC 367), were tested for their pathogen exclusion potential during infection with *S.* Typhimurium using differentiated colonic epithelial cells (Caco2) (Figure 1A). All organisms varied in their capacity to significantly (*p* < 0.045) reduce *Salmonella* adhesion. Of the five strains tested, *B. infantis* demonstrated the greatest capacity to significantly (*p* < 0.05) reduce *Salmonella* association with the epithelial cell and so was selected for further evaluation of potential molecular mechanisms underlying this attenuation.

### *B. infantis* altered adhesion of *S.* Typhimurium to colonic epithelial cells

The ability of *B. infantis* to displace, block, or outcompete *S.* Typhimurium was tested by altering the point of addition and the incubation time of both *B. infantis* and *S.* Typhimurium with differentiated colonic epithelial cells *in vitro* (Figure 1B). *B. infantis* addition to *Salmonella* infected epithelial cells, showed no effect on the pathogen’s ability to invade the host cell or decrease expansion of the infection, suggesting *B. infantis* was unable to displace already adhered *Salmonella* or reduce infection. In contrast, *B. infantis* added to epithelial cells prior to *S.* Typhimurium addition significantly (*p* < 0.05) reduced *S.* Typhimurium adhesion and invasion, as well as when the pathogen and probiotic cultures were added to epithelial cells concurrently. Interestingly, the co-incubation of *S.* Typhimurium and *B. infantis* for 60 min resulted in a significant (*p* < 0.05) increase in host association by *S.* Typhimurium compared to the other combination treatments.

The finding that *B. infantis* was host-protective only when added prior to or concurrently with *S.* Typhimurium suggests potential involvement of physical exclusion mechanisms that prohibits *S.* Typhimurium from gaining access to the host membrane for binding and invasion. To evaluate if this seemingly opposite result stemmed from pathogen-probiotic competition for host cell receptors, receptors important for *Salmonella* adhesion and invasion in Caco2 cells (HSP90, PPP1R12A, CTNN1A, ganglioside GD3, and ganglioside GM1) (Desai, 2011) were blocked using antibodies prior to addition of either *B. infantis* or *S.* Typhimurium (Supplementary Figure S1). Unlike the other receptors tested, blocking ganglioside GM1 on the host reduced *B. infantis* adhesion by 25% as compared to the unblocked control condition (*p* = 0.05) (Supplementary Figure S1), indicating *B. infantis* and *S.* Typhimurium may compete to bind this specific host receptor. Notably, the binding of *S.* Typhimurium and *B. infantis* to the same host ganglioside GM1 receptor, which regulates host inflammation cascades, resulted in diametrically opposed downstream expression patterns in epithelial cells (Supplementary Figure S2).

### Autocrine signaling loops and apoptotic pathways are regulated by association with *B. infantis*

The host ganglioside receptors, including GM1, are important modulators of the inflammatory response and involved in orchestrating the response to pathogen invasion (Park et al., 2023). The involvement of the ganglioside GM1 receptor in autocrine signaling, such as the IL-6 cascade, coupled with the observed affinity of *B. infantis* for this receptor led us to postulate that *B. infantis* association with human epithelial cells may initiate other host-protective signaling cascades. The canonical G-protein coupled receptor pathway (GPCR) was significantly induced (adj-*p* ≤ 0.04) in epithelial cells when incubated with *B. infantis* over 120 min, along with four GCPR ligands (CXCL1, CXCL2, CXCL3 and CCL20) and IL-6 that all showed significant induction (adj-*p* ≤ 0.00) (Table 1; Supplementary Table S1). Furthermore, genes downstream of IL6 signaling, which are under the positive transcriptional control of phosphorylated STAT3, were also induced (adj-*p* ≤ 0.00) (Table 2). Taken together, this collection of induced genes likely stimulated an autocrine signaling loop in the epithelial cell resulting from association with *B. infantis*.

**Table 1:** Differentially regulated GO gene sets (adj-*p* ≤ 0.21) in Caco2 cells when exposed to *B. infantis* and *S.* Typhimurium.

Name	Size of gene set	Genes regulated	NES	Adj-*p* value
Biological process				
Apoptotic mitochondrial changes	10	7	−2.15	0.02
Mitochondrion organization and biogenesis	43	20	−2.43	0.00
Oxygen and reactive oxygen species metabolic process	12	7	−2.05	0.05
Cellular component				
Coated vesicle membrane	13	3	−1.69	0.07
Contractile fiber	15	8	−1.70	0.07
Contractile fiber part	15	8	−1.71	0.07
Envelope	145	57	−1.70	0.07
Extracellular region	160	70	−1.68	0.07
Extracellular space	80	33	−1.85	0.02
Microbody	39	15	−1.67	0.07
Microbody membrane	12	8	−1.64	0.08
Microbody part	13	8	−1.68	0.07
Mitochondrial envelope	85	36	−1.96	0.01
Mitochondrial inner membrane	60	32	−2.33	0.00
Mitochondrial lumen	44	27	−2.26	0.00
Mitochondrial matrix	44	27	−2.17	0.00
Mitochondrial membrane	77	34	−1.96	0.01
Mitochondrial membrane part	50	36	−2.21	0.00
Mitochondrial part	128	59	−2.27	0.00
Mitochondrial respiratory chain	23	17	−1.90	0.01
Mitochondrial respiratory chain complex I	14	9	−2.01	0.01
Mitochondrial ribosome	22	18	−2.34	0.00
Mitochondrial small ribosomal subunit	11	9	−1.95	0.01
Mitochondrion	283	133	−2.22	0.00
NADH dehydrogenase complex	14	9	−1.98	0.00
Nucleolar part	12	9	−1.92	0.01
Nucleolus	91	39	−1.90	0.01
Organellar ribosome	22	18	−2.36	0.00
Organellar small ribosomal subunit	11	9	−1.97	0.01
Organelle envelope	145	57	−1.73	0.06
Organelle inner membrane	67	36	−2.43	0.00
Organelle lumen	341	95	−1.57	0.02

Name	Size of gene set	Genes regulated	NES	Adj-*p* value
Organelle membrane	243	85	−1.64	0.08
Peroxisomal membrane	12	8	−1.62	0.09
Peroxisomal part	13	8	−1.66	0.07
Peroxisome	39	15	−1.61	0.09
Proteasome complex	22	16	−1.62	0.09
Respiratory chain complex I	14	9	−2.02	0.01
Ribonucleoprotein complex	111	59	−1.92	0.01
Ribosomal subunit	20	16	−2.18	0.00
Ribosome	37	25	−2.36	0.00
Sarcomere	7	5	−1.65	0.08
Small ribosomal subunit	11	9	−1.95	0.01
Vesicle coat	13	3	−1.64	0.08
Molecular function				
Carbohydrate binding	21	9	−1.77	0.09
Carbon carbon lyase activity	16	8	−1.88	0.05
Carboxy lyase activity	12	7	−1.97	0.07
Cytokine activity	26	11	−1.72	0.11
Glycosaminoglycan binding	12	7	−1.71	0.11
Gtp binding	33	15	−1.93	0.08
Gtpase activity	67	15	−1.89	0.05
Guanyl nucleotide binding	33	15	−1.90	0.07
Heparin binding	10	7	−1.92	0.07
Hormone activity	10	5	−1.71	0.10
Oxidoreductase activity acting on NADH or NADPH	21	9	−1.85	0.06
Oxygen binding	9	4	−1.84	0.06
Pattern binding	13	8	−1.77	0.10
Ribonuclease activity	17	12	−1.78	0.10
Serine type endopeptidase inhibitor activity	15	8	−1.89	0.06
Transferase activity transferring alkyl or aryl other than methyl groups	25	11	−1.79	0.10

NES, Normalized enrichment score. A positive score indicates that the gene set was induced in Caco2 cells when exposed to *B. infantis* and *S.* Typhimurium, while a negative score indicates that the gene set was repressed.

**Table 2:** Top ten differentially regulated (adj-*p* ≤ 0) transcriptional regulons in Caco2 cells when exposed to *B. infantis*.

Gene set	Binding motif	Transcription factor	Remarks	Size of gene set	Genes regulated	NES	Adj *p*-value
V$CEBP_Q2_01	NTTRCNNAANNN	CEBPA: CCAAT/enhancer binding protein (C/EBP), alpha	Positive regulation by activated STAT3 (Numata et al., 2005), positive regulation by activity of Erk 1/2 (Radomska et al., 2006)	128	59	2.17	0.00
V$CDPCR1_01	NATCGATCGS	CUTL1: cut-like 1, CCAAT displacement protein (Drosophila)	Negatively regulates transcription of genes; repression is relieved by phosphorylation of CULTL1 by PKA (Michl et al., 2006) and PKC	53	19	2.04	0.00
V$CREB_Q2	NSTGACGTAANN	CREB1: cAMP responsive element binding protein 1	Activated by a variety of stimuli leading to phosphorylation of CREB1 mediated by PKA principally through GPCR signaling (Johannessen et al., 2004)	140	69	1.96	0.00
V$CRX_Q4	YNNNTAATCYCMN	CRX: cone-rod homeobox	SNPs in CRX are correlated to Crohn’s disease (Wellcome Trust Case Control Consortium, 2007)	104	50	2.05	0.00
V$NFKAPPAB_01	GGGAMTTYCC	NFKB RELA: v-rel reticuloendotheliosis viral oncogene homolog A	Activated by a diverse group of receptors which include cytokine receptors, T cell receptors, growth factor receptors and Toll like receptors (Mantovani, 2010)	95	46	1.97	0.00
V$PAX8_01	NNNTNNNGNGTGANN	PAX8: paired box gene 8	Activity is negatively regulated by glutathionylation of PAX8 (Cao et al., 2005)	16	11	1.94	0.00
V$OCT1_01	NNNNWTATGCAAATNTNNN	POU2F1: POU domain, class 2, transcription factor 1	Activity positively regulated by BRCA (Fan et al., 2002) and negatively regulated by Glucocorticoid-GCR complex (Kutoh et al., 1992)	103	54	2.27	0.00
V$OCT1_03	NNNRTAATNANNN	POU2F1: POU domain, class 2, transcription factor 1	Activity positively regulated by BRCA (Fan et al., 2002) and negatively regulated by Glucocorticoid-GCR complex (Kutoh et al., 1992)	93	40	2.05	0.00
V$SRF_Q4	SCCAWATAWGGMNMNNNN	SRF: serum response factor (c-fos serum response element-binding transcription factor)	This gene is the downstream target of many pathways; for example, the mitogen-activated protein kinase pathway (MAPK)	124	45	2.10	0.00
V$STAT3_02	NNNTTCCN	STAT3: signal transducer and activator of transcription 3 (acute-phase response factor)	This protein is activated through phosphorylation in response to various cytokines and growth factors including IFNs, EGF, IL5, IL6, HGF, LIF and BMP2 (O'Shea, 1997)	75	25	2.08	0.00

NES, Normalized enrichment score. A positive score indicates gene set induction in Caco2 cells, while a negative score indicates repression.

**Figure 2:**
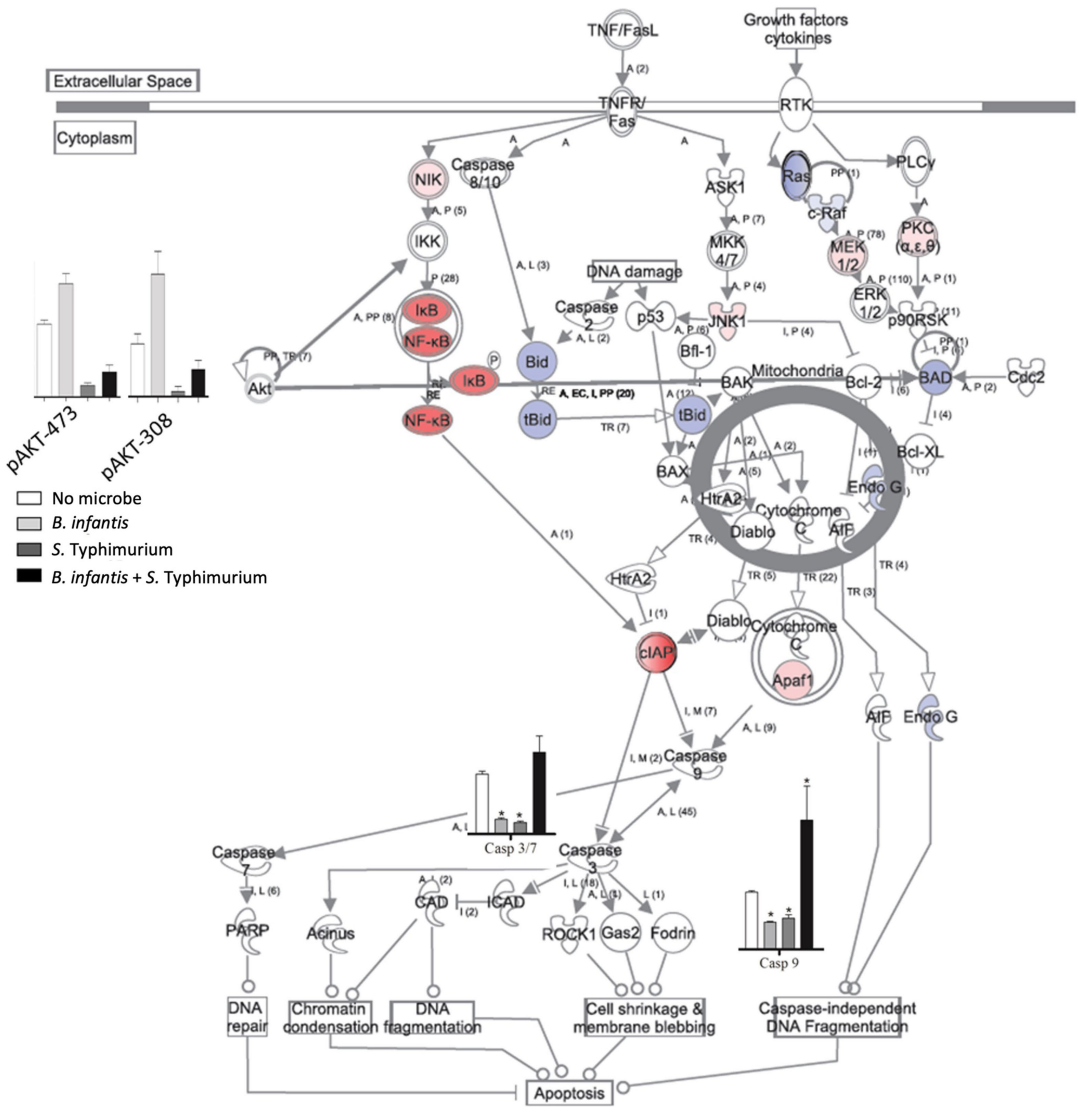
Log2 ratios of differentially expressed genes (adj-*p* ≤ 0.1); caspase 8, 9, 3/7 activity and phosphorylation status of Akt in epithelial cells when they are exposed to *B. infantis*. For gene expression data, red represents induction while blue represents repression of genes when epithelial cells were exposed to *B. infantis*.

The notable effect of *B. infantis* association on host autocrine signaling led us to examine the potential probiotic modulation of other epithelial cell pathways related to infection. Apoptotic signaling pathway in epithelial cells generally showed repression of cell death pathway after 120 min of *B. infantis* exposure (Figure 2), as well as in the presence of added *S.* Typhimurium with *B. infantis* (Supplementary Figure S3). Relatedly, *B. infantis* treatment significantly (adj-*p* < 0.05) lowered the overall basal caspase expression in epithelial cells and blocked the increase in *Salmonella*-mediated caspase activity. This observation of caspase regulation at the gene expression level was further confirmed via measurement of Akt phosphorylation status on ser473 and thr308 (Supplementary Figure S4). Together these data indicate regulation of host apoptotic signaling is an important factor that determines the success of *Salmonella* infection and the ability of *B. infantis* to alter the course of infection.

### *S.* Typhimurium overrides *B.* protective effects in the host

**Figure 3:**
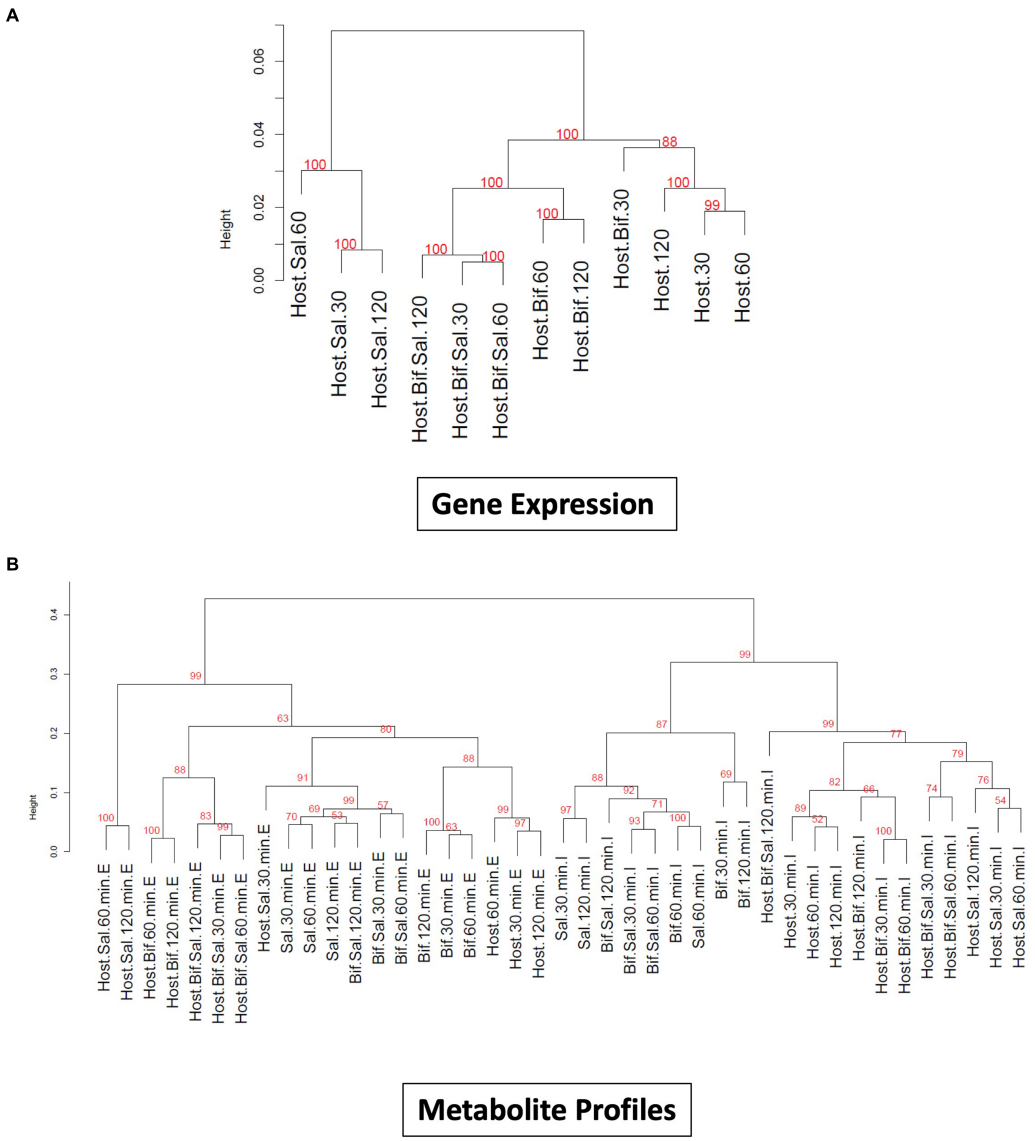
**(A)** Dendogram visualizing similarity between global gene expression profiles of Cac02 cells. The number at each cluster edge represents Approximately Unbiased % *p* value estimated by multiscale bootstrap resampling 1,000 times. (Host=Cac02 cells incubated with no microbes, Host+Bif=Cac02 cells incubated with *B. infantis*, Host+Sal=Cac02 cells incubated with *S.* Typhimurium, Host+Bif+Sal=Cac02 cells simultaneously incubated with 1:1 ratio of *B. infantis* and *S.* Typhimurium. The time in minutes represent the time after infection at which the gene expression was determined. **(B)** Dendogram constructed by hierarchical clustering of small metabolite profile of all the host microbe interaction samples. The number at each cluster edge represents Approximately Unbiased % *p* value estimated by multiscale bootstrap resampling 1,000 times (Host = Cac02, Bif = *B. infantis*, Sal = *S.* Typhimurium). The time in minutes represent the time after infection at which the metabolite profile was determined. “E” represents the extracellular metabolite profile from culture supernatant, while “I” represent the intracellular metabolite profile of all the cells in co-culture.

The incubation of epithelial cells with *B. infantis* alone resulted in the induction of multiple protective effects for the epithelial cell, but it was unclear if these same pathways would remain induced in the presence of *S.* Typhimurium. Hierarchical clustering of averaged global gene expression profiles of the different treatments as a time series indicated that the transcriptome of epithelial cells infected with *S.* Typhimurium and *B. infantis* simultaneously is more like that of the solo *B. infantis* treatment than to the transcriptome of *S.* Typhimurium infected cells (adj-*p* < 0.01) (Figure 3A). This ability of *B. infantis* to modulate host cell expression despite the addition of *S.* Typhimurium was not consistent across all the genes or pathways examined in this study. Genes annotated in the GO category of cell junctions that were induced by *B. infantis* alone were contrastingly significantly repressed (adj-*p* ≤ 0.13) in presence of *S.* Typhimurium (Table 3; Supplementary Table S2). The ability of *Salmonella* to supersede host-protective effects from *B. infantis* incubation suggests there are more multi-factorial mechanisms that alter gene expression regulation that allows the pathogen to have a larger contributing factor for expression regulation as compared to the probiotic interaction.

**Table 3:** GO gene sets that were differentially regulated (adj-*p* ≤ 0.21) in Caco2 cells when exposed to *B. infantis*.

Name	Size of gene set	Genes regulated	NES	Adj-*p* value
Biological process				
Aerobic respiration	15	9	−1.72	0.21
Apoptotic mitochondrial changes	10	5	−1.78	0.21
Base excision repair	13	6	−1.77	0.21
Cellular component disassembly	22	9	−1.74	0.21
Cellular protein complex disassembly	10	4	−1.70	0.20
Coagulation	20	8	−1.72	0.20
DNA catabolic process	16	10	−1.97	0.13
Electron transport	34	14	−1.83	0.20
Embryonic development	34	18	1.99	0.12
Embryonic morphogenesis	10	8	1.90	0.19
Mitochondrion organization and biogenesis	43	19	−2.35	0.00
Negative regulation of cell differentiation	15	9	1.81	0.16
Oxygen and reactive oxygen species metabolic process	12	8	−1.87	0.18
Pattern specification process	19	10	1.84	0.18
Protein amino acid autophosphorylation	22	13	1.83	0.15
Protein folding	50	26	−1.69	0.20
Regulation of body fluid levels	26	10	−1.81	0.20
Response to toxin	7	4	−1.96	0.10
Ribosome biogenesis and assembly	12	8	−1.67	0.21
rRNA processing	9	3	−1.77	0.20
Transcription from RNA polymerase iii promoter	17	7	−1.68	0.20
Cellular component				
Adherens junction	12	6	1.76	0.13
Basolateral plasma membrane	22	9	1.78	0.16
Cell junction	48	19	1.93	0.05
Cell matrix junction	10	5	1.70	0.13
Envelope	145	64	−1.92	0.01
Intercellular junction	39	14	1.75	0.11
Kinesin complex	11	7	1.64	0.18
Mediator complex	8	5	−1.71	0.03
Membrane enclosed lumen	341	133	−1.84	0.01
Microbody	39	18	−1.78	0.02
Mitochondrial envelope	85	46	−2.30	0.00
Mitochondrial inner membrane	60	41	−2.67	0.00
Mitochondrial lumen	44	24	−2.63	0.00
Mitochondrial matrix	44	24	−2.55	0.00
Mitochondrial membrane	77	44	−2.33	0.00
Mitochondrial membrane part	50	25	−2.64	0.00
Mitochondrial part	128	72	−2.72	0.00
Mitochondrial respiratory chain	23	16	−2.26	0.00
Mitochondrial respiratory chain complex I	14	10	−2.34	0.00
Mitochondrial ribosome	22	19	−2.78	0.00
Mitochondrial small ribosomal subunit	11	10	−2.17	0.00
Mitochondrion	283	161	−2.60	0.00
NADH dehydrogenase complex	14	10	−2.33	0.00
Nucleolar part	12	9	−2.09	0.00
Nucleolus	91	31	−1.93	0.01
Organellar ribosome	22	19	−2.79	0.00
Organellar small ribosomal subunit	11	10	−2.19	0.00
Organelle envelope	145	64	−1.96	0.00
Organelle inner membrane	67	39	−2.67	0.00
Organelle lumen	341	106	−1.86	0.01
Organelle membrane	243	75	−1.84	0.01
Peroxisome	39	18	−1.79	0.02
Proteasome complex	22	16	−1.83	0.01
Proton transporting two sector ATpase complex	15	8	−1.71	0.04
Respiratory chain complex I	14	10	−2.35	0.00
Ribonucleoprotein complex	111	46	−2.41	0.00
Ribosomal subunit	20	16	−2.70	0.00
Ribosome	37	22	−2.70	0.00
Small ribosomal subunit	11	10	−2.15	0.00
Molecular function				
Aldo keto reductase activity	7	6	−1.68	0.12
Antioxidant activity	14	12	−1.64	0.15
Cytochrome c oxidase activity	12	8	−1.71	0.12
Damaged DNA binding	18	6	−1.74	0.10
Deoxyribonuclease activity	16	9	−1.69	0.13
Electron carrier activity	58	28	−1.99	0.01
Endonuclease activity	20	10	−1.78	0.08
Endonuclease activity GO 0016893	10	6	−1.82	0.06
Endoribonuclease activity	11	7	−1.92	0.03
Exonuclease activity	14	9	−1.82	0.06
Hormone activity	10	4	−1.71	0.13
Methyltransferase activity	29	13	−1.67	0.13
Nuclease activity	37	22	−2.05	0.01
Oxidoreductase activity	189	91	−2.05	0.01
Oxidoreductase activity acting on NADH or NADPH	21	13	−2.13	0.00
Ribonuclease activity	17	10	−2.15	0.00
RNA polymerase activity	13	7	−1.93	0.02
Serine type endopeptidase activity	22	9	−1.71	0.11
Serine type endopeptidase inhibitor activity	15	6	−1.68	0.12
Structural constituent of ribosome	73	41	−2.05	0.01
Transferase activity transferring alkyl or aryl other than methyl groups	25	16	−1.88	0.03
RNA polymerase ii transcription factor activity enhancer binding	9	5	1.92	0.14

NES, Normalized enrichment score. A positive score indicates that the gene set was induced in Caco2 cells when exposed to *B. infantis*, while a negative score indicates that the gene set was repressed.

### *S.* Typhimurium virulence factors were induced by *B. infantis* during co-culture

One mechanism that may explain the ability of *S.* Typhimurium to override the potential host-protective mechanisms of *B. infantis* is the induction of *Salmonella* virulence factors in response to pathogen-probiotic co-incubation. A time series gene expression experiment (30, 60, and 120 min) (Figure 1B), revealed genes coding for both Type III Secretion Systems (T3SS) (SPI1 T3SS (adj-*p* ≤ 0), SPI2 T3SS (adj-*p* ≤ 0.04)), and effectors for both T3SSs (adj-*p* < 0.05), were significantly induced in the *B. infantis* co-culture condition, independent of epithelial cells (Supplementary Figure S5). The increased expression of the T3SS and its component parts in co-culture with *B. infantis* suggests *B. infantis* co-operatively potentiated the virulence of *S.* Typhimurium via shared environmental signals. One mechanism employed by *S.* Typhimurium to sense the local environment is the use of two-component systems, which have been linked to regulating virulence in fellow enteric pathogen *E. coli* (Reading et al., 2009). Virulence related two-component systems (Qse and SsrAB) in *Salmonella* (Merighi et al., 2009) were induced in the presence of *B. infantis*, irrespective of host cell presence (adj-*p* ≤ 0.09) (Supplementary Tables S3, S4).

The significant induction of both the T3SS and related two-component systems in the presence of *B. infantis* suggests that this probiotic organism primed virulence in *Salmonella* via multiple routes as well as being responsive to environmental cues during co-incubation. These environmental cues not only altered gene expression of *Salmonella*, but of *B. infantis* as well (Supplementary Table S5). Intriguingly, in the presence of *Salmonella*, *B. infantis* notably regulated genes that are conserved only in the subspecies *infantis* and not in divergent subspecies *longum* (Supplementary Figure S6; Supplementary Table S6). Regulation of this gene set that is horizontally acquired and unique to subspecies *infantis* suggests the effects of *B. infantis* observed here are specific to this taxonomic grouping. The co-regulation of *Salmonella* virulence factors and of *B. infantis* horizontally acquired gene sets, in conjunction with the noted importance of incubation order, highlight changes in the local environment are integral to probiotic-pathogen interaction outcome, brining into focus that bacterial crosstalk via small molecules is important in enteric infection (Table 4).

**Table 4:** Differentially regulated pathways (adj-*p* ≤ 0.05) in Caco2 cells when exposed to *B. infantis*.

Gene set	Size of gene set	Genes regulated	NES	Adj-*p* value
KEGG pathway				
Parkinsons disease	92	59	−2.78	0
Oxidative phosphorylation	96	63	−2.75	0
Proteasome	39	27	−2.55	0
Alzheimer’s disease	118	58	−2.3	0
Glutathione metabolism	38	25	−2.16	0
Huntingtons disease	140	76	−2.14	0
Spliceosome	104	54	−2.03	0
Drug metabolism other enzymes	24	11	−1.89	0.02
Pyrimidine metabolism	72	26	−1.88	0.02
Ribosome	75	42	−1.86	0.02
Peroxisome	60	29	−1.85	0.02
RNA polymerase	27	17	−1.82	0.03
Metabolism of xenobiotics by cytochrome P450	32	20	−1.82	0.03
DNA Replication	32	18	−1.82	0.03
Antigen processing and presentation	37	20	−1.82	0.03
Type I diabetes mellitus	12	9	−1.77	0.04
Arachidonic acid metabolism	21	14	−1.71	0.05
Autoimmune thyroid disease	11	8	−1.71	0.05
Notch signaling pathway	35	18	1.83	0.05
Phosphatidylinositol signaling system	45	22	1.89	0.04
Hedgehog signaling pathway	27	7	1.9	0.04
Adherens junction	63	38	1.93	0.04
Focal adhesion	126	57	1.94	0.05
Biocarta pathway				
Proteasome complex	18	13	−1.97	0.01
Intrinsic prothrombin activation pathway	14	9	−1.74	0.04
Phospholipids as signaling intermediaries	20	8	1.86	0.04
Multiple antiapoptotic pathways from IGF-1R signaling lead to BAD phosphorylation	19	8	1.89	0.04
GPCR pathway	25	10	1.89	0.04
IL 6 signaling pathway	19	8	1.91	0.04
PDGF signaling pathway	27	10	1.92	0.04
Thrombopoietin (TPO) signaling pathway	18	9	1.93	0.04
EGF signaling pathway	27	10	1.94	0.05
Insulin signaling pathway	18	8	1.96	0.05

NES, Normalized enrichment score. A positive score indicates that the gene set was induced in Caco2 cells when exposed to *B. infantis*, while a negative score indicates that the gene set was repressed.

### Small molecules mediate gene expression and bacterial binding

Results from this study consistently demonstrate that multiple mechanisms mediate crosstalk that mediates a triad of cellular behavior between *S.* Typhimurium, *B. infantis,* and the epithelial cell that change the phenotype of metabolism and interaction. Induction of *S.* Typhimurium virulence genes, competition for host receptor GM1, and total gene expression changes observed in this study indicate that the progression of *S.* Typhimurium infection is affected by the activity of both *B. infantis* and the epithelial cells in more complex ways than simple bacterial competition for host receptor binding. One mechanism by which *S.* Typhimurium stages a successful infection and that may allow for probiotic bacterial competition is through a multifaceted metabolic coup.

Small molecules are increasingly being associated with bacterial adhesion on epithelial cells (Ghorashi and Kohler, 2020; Lin et al., 2020). Modification via glycosylation of proteins, which also play a role in mediating of bacterial binding (Barboza et al., 2012; Arabyan et al., 2016) is also a route that likely leads to cross-talk. In complement to the gene expression profiles, small metabolites were measured from the same epithelial cell treatment used for gene expression. Metabolite profiling using GC/MS identified 294 compound peaks using the binbase database (Fiehn et al., 2005), out of which 110 GC/MS peaks were assigned an identification (Figure 3). Hierarchical clustering of intracellular and extracellular small molecules (Figure 3B) followed a similar pattern to that of the global gene expression profiles (Figure 3A), where solo *S.* Typhimurium infection cluster separately from the control, solo *B. infantis*, and mixed culture treatments. Hierarchical clustering based on log_2_ peak areas of all compounds revealed two clusters for the extracellular metabolites and another for intracellular metabolites (adj-*p* < 0.01) (Figure 3B). Within the extracellular metabolite cluster, epithelial cells co-infected with *Salmonella* and *B. infantis* together clustered with epithelial cells treated with *B. infantis* alone (adj-*p* > 0.0.05). Epithelial cells infected with *S.* Typhimurium formed a unique cluster (adj-*p* < 0.01) at 60 and 120 min, demonstrating the divergence in the trajectory of infection progression. Generally, the gene expression profile and the extracellular metabolome of epithelial cells co-infected with *S.* Typhimurium and *B. infantis* were more like those of cells treated with *B. infantis* than with *S.* Typhimurium.

In summary, the gene expression and metabolic profiles paint a complex picture of a locally nuanced probiotic effect, wherein the association of *B. infantis* potentiates host-protective effects but the positive modulation is in part overridden by *S.* Typhimurium. The nuanced nature highlights the continuous observation that local environment mediates the host response as well as incubation with *B. infantis* alone versus with the *B. infantis*/*S.* Typhimurium combination. These orthogonal and consistent observations led us to hypothesize that the presence of *S.* Typhimurium and epithelial cells must also alter the activity of *B. infantis.*

### The course of *S.* Typhimurium infection was altered by the presence of *B. infantis*

**Figure 4:**
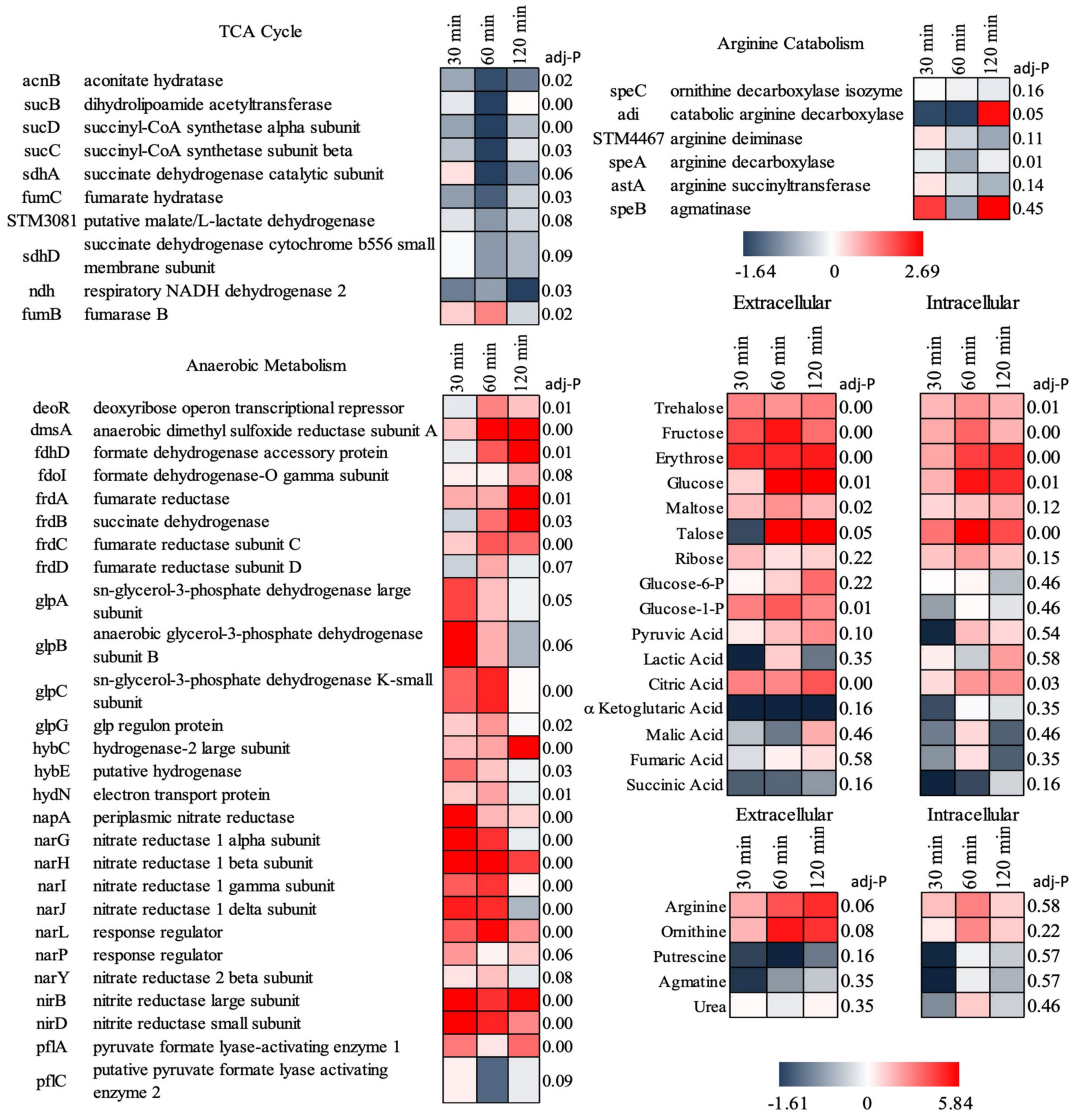
Log2 ratios of gene expression intensities of *S.* Typhimurium and selected small metabolite peak areas when Cac02 cells were treated with either *S.* Typhimurium alone or with a co-culture of *S.* Typhimurium and *B. infantis*. Induction of genes represent that the gene was induced in *Salmonella* in presence of *B. infantis* as compared to its absence.

Supporting this potential involvement of metabolic control in probiotic interaction was the marked shift in *Salmonella*’s metabolism in response to the co-culture partners. The addition of *B. infantis* to the host/*Salmonella* co-culture resulted in the repression of arginine catabolism genes in *S.* Typhimurium (Figure 4). This repression was likewise reflected in the small molecule profiles, where *B. infantis* addition led to the accumulation of arginine and ornithine with corresponding depletion of putrescine. In contrast, depletion of arginine was observed from *Salmonella/*epithelial cell co-culture in absence of *B. infantis.* Arginine accumulated when *B. infantis* was present, suggesting an increased substrate pool for nitric oxide (NO) production and provides a mechanism leading to hypervirulent *Salmonella* (Heithoff et al., 2012). The major pathway for NO metabolism is the stepwise oxidation to nitrite (NO_2_^−^) and nitrate (NO_3_^−^) (Lundberg et al., 2008). In presence of *B. infantis*, nine genes related to nitrate and nitrite reduction were significantly induced (adj-*p* ≤ 0) in *Salmonella* (Figure 4, “Anaerobic Metabolism”). The required genes in the pathway for synthesis of cofactors necessary for the activity of nitrate and nitrite reductases were also significantly induced in *Salmonella* co-cultured with *B. infantis* (adj-*p* ≤ 0) (Table 5, “Biosynthesis of Cofactors-Prosthetic Groups-And Carriers-Molybdopterin”). *B. infantis*-mediated induction of nitrate and nitrite reductases when co-cultured with *Salmonella* only occurred in the presence of epithelial cells, indicating induction of nitrate and nitrite reductases in *Salmonella* was likely a downstream effect of the epithelial cell metabolism, perhaps nitric oxide metabolism, but highlights the complexity of the metabolic interactions between the cellular triad, which led us to investigate energy cycling.

**Table 5:** Differentially regulated gene sets (adj-*p* ≤ 0.09) in *S.* Typhimurium when incubated with epithelial cells as compared to when incubated with epithelial cells and *B. infantis* for 120 min.

Gene set	Size of gene set	Genes regulated	NES	Adj-*p* value
Biosynthesis of cofactors-prosthetic groups-and carriers-molybdopterin	9	6	1.60	0.08
Cog J-translation	170	107	2.51	0.00
Cog K-transcription	246	96	1.76	0.04
DNA metabolism-DNA replication-recombination- and repair	119	54	1.61	0.09
Energy metabolism-anaerobic	66	26	2.21	0.00
Genes induced by QSE two component system	44	29	1.63	0.09
Glycan biosynthesis-LPS biosynthesis	27	13	1.74	0.05
Membrane transport-protein export	16	11	1.80	0.03
Protein fate-protein folding and stabilization	34	11	1.60	0.09
Protein fate-protein modification and repair	20	11	1.64	0.09
Protein synthesis-ribosomal proteins-synthesis and modification	61	46	1.94	0.01
Protein synthesis-translation factors	28	19	1.88	0.01
Protein synthesis-tRNA And rRNA base modification	28	17	2.27	0.00
Purines-Pyrimidines- Nucleosides-and Nucleotides-2-Deoxyribonucleotide metabolism	10	4	1.58	0.09
SPI-3	9	4	1.60	0.09
Transcription-RNA processing	7	6	1.60	0.08
Mobile and extrachromosomal element functions-prophage functions	143	64	−2.41	0.00
Mobile and extrachromosomal element functions-plasmid functions	28	24	−2.01	0.00
Membrane transport-ABC transporters	174	57	−1.99	0.00
HGT-high %GC	70	46	−2.60	0.00
Genes with %GC greater than 60	196	100	−2.54	0.00
Energy metabolism-TCA cycle	35	8	−1.65	0.09
Cellular processes-DNA transformation	30	26	−1.99	0.00
Biosynthesis of cofactors-prosthetic groups-and carriers-biotin	8	4	−1.89	0.01

NES, Normalized enrichment score. A positive score indicates that the gene set was induced in *S.* Typhimurium when incubated with Caco2 cells and *B. infantis* together, while a negative score indicates that the gene set was repressed.

Further, addition of *B. infantis* to the host/*S.* Typhimurium co-culture also repressed *Salmonella* genes encoding for the TCA cycle (adj-*p* ≤ 0.09), and contrastingly induced genes necessary for anaerobic metabolism (adj-*p* ≤ 0) (Table 5; Figure 4). Genes necessary for mitochondrial respiratory chain function were also repressed in epithelial cells with the addition of *B. infantis* into the host/pathogen co-culture (Tables 2, 3). Addition of *B. infantis* resulted in repression of genes necessary for respiration in both epithelial cells and *S.* Typhimurium. Repression of respiration was also reflected in the small metabolite pool of the probiotic-pathogen co-culture with epithelial cells. Incubation of *S.* Typhimurium and host cells with *B. infantis* resulted in a significant accumulation of glucose, fructose, trehalose, and maltose (adj-*p* < 0.05) extracellularly and intracellularly. Glucose-6P, pyruvate, and citrate also significantly accumulated (adj-*p* ≤ 0.01) in the same conditions, while the remaining TCA metabolites (α-ketoglutarate, malate, fumarate, and succinate) did not significantly change, though a clear reduction was observed. *B. infantis* addition to the host/pathogen co-culture reduced the carbon flux through glycolysis and the TCA cycle, while the presence of *Salmonella* alone with epithelial cells induced host genes related to glycolysis that would influence the overall energy flux in each cell. The combination of small metabolite phenotyping and related gene expression reveal an intricate web of control wherein three-way crosstalk between host, probiotic, and pathogen drives shared metabolism and in turn either mitigates or exacerbates infection in a local context-dependent manner.

## Discussion

This study examined the ability of *B. infantis* to attenuate *S.* Typhimurium infection in an *in vitro* model and further characterized underlying microbe-microbe and host–microbe interactions. While *B. infantis* reduced adhesion and invasion to host cells, this significant mitigation of *S.* Typhimurium was only seen when *B. infantis* was added prior to or concurrently with *Salmonella*. *B. infantis* was unable to displace already adhered *S.* Typhimurium bacteria, suggesting that one mode of exclusion may be competition for specific host receptors, wherein early access to the receptor by *Bifidobacteria* effectively blocks *Salmonella* adherence.

The probiotic-pathogen pair in this work share an affinity for the host ganglioside GM1 receptor but not other known bacterial receptors commonly used by *Salmonella*. The observed interaction of *B. infantis* with the GM1 receptor may be associated with the co-evolution of *B. infantis* and breast milk (Underwood et al., 2015). Milk across species contain ganglioside binding motifs (Cacho and Lawrence, 2017; Tan et al., 2020) and *B. infantis* is genetically equipped to consume the complex oligosaccharides in breast milk (HMOs), making physical interactions with milk components evolutionarily advantageous to *B. infantis* (Lawson et al., 2020). In the context of the host receptors, binding to GM1 receptors in the gut epithelia by *B. infantis* may serve as a signal to the immune system (Kimata, 1994; Mou et al., 2022) and such immunostimulatory effects of bifidobacteria have been thoroughly reported by others in the literature (Ruiz et al., 2005; Henrick et al., 2021; Lueschow et al., 2022). Host ganglioside GM1, alongside other ganglioside receptors, modulate the expression of interleukins including pleiotropic and anti-inflammatory IL6 (Mou et al., 2022). Further supporting the strain-specific effects of probiotic and pathogen adhesion to epithelial cells, previous work in *Salmonella* has shown the host gut epithelial can recognize strain level variation and such recognition influences downstream expression (Salerno-Gonçalves et al., 2018; Salerno-Goncalves et al., 2019). As seen in this study, IL6 expression was induced in epithelial cells in the presence of *B. infantis* alone, potentiating host-protective mechanisms. The addition of *Salmonella* appears to dampen protective *B. infantis* immunomodulation activity, suggesting host modulation by *B. infantis* could be a selective effect, overridden by the exerted pressures of enteric pathogens, as shown with the IL6/NFkB expression pattern observed here.

Underscoring the ability of *Salmonella* to obstruct immunomodulatory properties of *B. infantis*, incubation of epithelial cells with *B. infantis* alone induced the transcription of NFkB related cytokines: IL6, CXCL1, CXCL2, CXCL3 and CCL20. Contrastingly, genes for these cytokines were repressed when *Salmonella* was added to the model. *Salmonella* interferes with host signal transduction, including pathways for producing these cytokines, by secreting a vast array of effector molecules directly into the host cytosol (Agbor and McCormick, 2011). One of these secreted effectors, AvrA, blocks NFkB activation by deubiquination of IkBα (Ye et al., 2007). Deubiquination of IkBα leads to the repression of target genes in the NFkB pathways, including IL6, a trend that was observed both in this study and previously by Ye et al. (2007). The repression on NFkB related cytokine genes in the present study indicates that *Salmonella* blocked the *B. infantis*-mediated induction of NFkB, likely through AvrA activity, which was induced in the presence of *B. infantis*.

Though *Salmonella* was able to negate some positive cytokine induction in the host from *B. infantis* interactions, host gene expression data for ROS production, cellular respiration, and mitochondrial biogenesis in this work reveals *B. infantis* may nevertheless modulate other host activity to reduce enteric infection. *B. infantis* in part mitigated *Salmonella* pathogenesis through modulation of mitochondrial dysfunction and related metabolic product ROS in epithelial cells. A variety of pathogens can induce cell death by causing mitochondrial dysfunction in the host (Rudel et al., 2010; Ashida et al., 2011; Lee et al., 2015; Ashida et al., 2021), which is induced by caspase 9 activation and is mediated through cytochrome c release in the host cytoplasm (Brentnall et al., 2013). Other work from our group has also correlated the induction of genes involved in production of ROS, respiration, and mitochondrial biogenesis to increased caspase 9 and caspase 3/7 activation during *Salmonella* infection (Shah et al., 2014), while caspase 8 and caspase 9 activity have been shown to increase in response to excess ROS (Fink and Cookson, 2007; Man et al., 2013; Shah et al., 2014; Hefele et al., 2018). Previous work has shown that mitochondrial production of ROS in the gut gives *Salmonella* a selective advantage, as ROS can react with luminal sulfur compounds to form tetrathionate, which can be used by *Salmonella* to respire, giving it a competitive edge over fermenting gut microbes (Winter et al., 2010). The results from this study suggest *B. infantis* may block ROS production in the gut through repression of host genes and pathways needed for aerobic respiration and functioning of the electron transport chain. Such a mechanism could contribute to the *Bifidobacteria-*mediated resistance to *Salmonella* observed here and also previously seen in mice (Shu et al., 2000; Huang and Huang, 2021).

As evidenced by the utilization of host-produced ROS detailed above, *S.* Typhimurium responds to and regulates the metabolic environment of the gut lumen (Das et al., 2010; Agbor and McCormick, 2011; Gart et al., 2016; Herrero-Fresno and Olsen, 2018). Manipulation of the gut metabolic environment by *S.* Typhimurium provides a competitive advantage to this pathogen in the crowded gastrointestinal microbial landscape (Taylor and Winter, 2020). Intriguingly, based on the metabolic profile clustering and more specific changes in select metabolites, there was a three-way, metabolically mediated, cross-talk between host, pathogen, and probiotic.

*Bifidobacteria infantis* prevented catabolism of arginine by *S.* Typhimurium, leading to arginine accumulation, which feeds increased host NO production (Wu et al., 2021). The increase in host NO production is supported by the significant induction of *Salmonella* genes necessary for nitrate and nitrite respiration, both products of NO metabolism. The mitigation of arginine metabolism in *Salmonella* by *Bifidobacteria* feeds host NO production, which then induces *Salmonella* to utilize nitrate and nitrite respiration pathways. The crosstalk between probiotic and pathogen observed here begins to explain the observations from the co-incubation condition, wherein *S.* Typhimurium appeared to have no change in, or even slightly increased, pathogenic fitness derived from the prebiotic’s presence.

The complex probiotic-pathogen interaction observed in this study was dependent on the local metabolic environment, as evidenced by the modulation of NO metabolism with gene expression and supported by small metabolite profiles. The extracellular metabolome of *S.* Typhimurium infected epithelial cells was markedly different than that of the *B. infantis* treated or concurrent *B. infantis* and *S.* Typhimurium infected cells, suggesting local metabolic control by *B. infantis* may be central to its probiotic efficacy or lack thereof. *Bifidobacterium infantis* was able to alter the metabolic environment of the host cells and maintained some of this metabolic control even in the presence of *S.* Typhimurium, as indicated by the hierarchical clustering of metabolic profiles. Such data support that metabolic control is one route by which probiotics may potentiate host protective effects, but simultaneously suggests such protective mechanisms are highly dependent on pathogenic metabolic abilities, further demonstrating with gene expression changes, protein alteration, and metabolites modulate the local environment.

In conclusion, *B. infantis* exerted protective effects against *Salmonella* in a colonic cell model through physical competition for the host ganglioside GM1 receptors; through repression of host cell death pathways, via the modulation of caspase activities; and metabolic shifts that change the energy balance and redox conditions that impact virulence capacity. The results of this work suggest *B. infantis* retains some but not all probiotic properties in the face of *Salmonella* infection, but follow-up work in animal models will be necessary to confirm these *in-vitro* observations with the additional complexity of the gut microbiome. In our model, these host-protective activities were contingent on incubation order, as *B. infantis* was not able to repress caspase activity when added after or concurrently with *Salmonella.* Notably, the mixed effect of *Bifidobacteria* addition on *Salmonella* pathogenicity extended to the modulation of bacterial and host metabolism. Probiotic co-incubation resulted in the repression of arginine catabolism by *Salmonella* and drove host NO production, ultimately leading the pathogen to regulate gene expression to efficiently utilize host-produced nitrate and nitrite. The finding that *Bifidobacteria* alter the metabolic activity of both the pathogen and the host supports that probiotic mechanisms are both nuanced and contextually driven. Further mechanistic research on this probiotic-pathogen pairing and others is crucial to understanding if these findings are specific to this combination or a more widespread phenomenon across probiotic-pathogen interactions; however, collectively, this study indicates that a complex relationship between the cellular triad of host, pathogen, and probiotic wherein the stage of probiotic addition, as a prophylactic or treatment, may ultimately dictate infection outcomes.

## Materials and methods

### Cell culture and bacterial strains

Colonic epithelial (Caco2) cells were obtained from ATCC (HTB-37, Manassas, VA) and cultured as per ATCC’s protocols and previously described (He et al., 2013; Arabyan et al., 2016). All the cells used in the assay were between passage numbers 22–30. In brief, cells were plated at a density of 10^5^/cm^2^ in either a T75 or a 96-well plate. Cells were maintained in DMEM/High Modified (Thermo Scientific, Rockford, IL) with 16.6% fetal bovine serum (FBS) (HyClone Laboratories, Logan, UT), non-essential amino acids (Thermo Scientific, Rockford, IL), 10 mM MOPS (Sigma, St. Louis, MO), 10 mM TES (Sigma), 15 mM HEPES (Sigma) and 2 mM NaH_2_PO_4_ (Sigma). Cells were considered to be differentiated 14 days post confluence (Ouwehand and Salminen, 2003), and used for the adhesion assays, caspase assays and gene expression. Bacterial cells were grown as described in Supplementary Table S7.

### Determination of adherence and invasion

The ability of specific bacteria to block *Salmonella* binding to epithelial cells was tested by determining the changes in the amount of intestinal epithelial cells associated *Salmonella* in presence of specific bacteria as previously described (He et al., 2013; Shah et al., 2014; Park et al., 2016; Chen et al., 2017). The amount of total host associated *Salmonella* was determined by qPCR as described below. The amount of total invaded *Salmonella* in the host were determined by the gentamicin protection assay (Arabyan et al., 2016) with modifications as described below.

Epithelial cells were cultured as described earlier in a 96-well plate (He et al., 2013; Shah et al., 2014; Park et al., 2016; Chen et al., 2017). The bacteria were used for the adhesion assays after two transfers. Bacterial cells were collected from 2 mL of media after growth for 14 h, washed twice with an equal volume of PBS, and re-suspended at ~10^8^ cfu/mL, in DMEM/High modified with 1X non-essential amino acids, 10 mM MOPS, 10 mM TES, 15 mM HEPES and 2 mM NaH_2_PO_4_ but without the FBS. Epithelial cells were infected with either *Salmonella* alone or *Salmonella* in conjunction with other bacteria in a final volume of 50 μL at an MOI (multiplicity of infection) of 1:1000. The ratio of *Salmonella* with other bacteria was 1:1 and incubated with epithelial for 60 min. The bacterial cell suspension was aspirated, and the Caco2 monolayer was washed thrice with 200 μL of Tyrode’s (140 mM NaCl, 5 mM KCl, 1 mM CaCl_2_, 1 mM MgCl_2_, 10 mM glucose, 10 mM sodium pyruvate, 10 mM HEPES, pH 7.4) to remove non-adhered bacterial cells from the monolayer. DNA extraction buffer (AEX Chemunex, France) (50 μL) was used to lyse the monolayer and the bacteria associated with the host, and incubated at 37°C for 15 min followed by 95°C for 15 min. Resulting cell lysate was used to determine the number of bacteria associated with the Caco2 cells (Desai et al., 2008). Quantitative analysis was done using qPCR with a CFX 96 Real Time System (BioRad, Hercules, CA). Reactions were performed in a final volume of 25 μL including 1 μL of cell lysate, 100 nM of PCR primers and iQ SYBR Green Supermix (BioRad, Hercules CA) as per manufacturer’s instructions. The primers used for the amplification are listed in Supplementary Table S9. The reaction parameters consisted of denaturation step at 95°C for 5 min, followed by 40 cycles of denaturation, annealing and extension at 95°C for 15 s, 56°C for 30 s, 72°C for 30 s, respectively, and a final extension at 72°C for 1 min. The product was verified using a melt curve analysis from 50°C to 95°C with a transition rate of 0.2°C/s. The number of bacterial cells and Caco2 cells present in each well were estimated using a standard curve of C_T_ *Vs* Log_10_ cfu and number of bacteria per Caco2 was calculated. The data were mean normalized relative to control wells, which were incubated with *Salmonella* alone. The experiment was done in four replicates. Differences in the mean due to treatment were tested by one way ANOVA followed by a means comparison to control using Dunnett’s multiple comparison test.

The amount of total invaded and adhered bacteria was determined using the gentamicin protection assay previously described (Chen et al., 2017). The infected cells were incubated for 60 min at 37°C with 5% CO_2_. Upon incubation, the media was aspirated, and cells were washed three times with Tyrode’s buffer. Invaded bacteria were estimated by incubating the cells with 100 μg/mL gentamicin for two hours at 37°C with 5% CO_2_ to kill bacteria adhered or outside the Caco2 cells. To determine total host associated bacteria, cells were incubated with cell culture media without any antibiotic. Cells were again washed three times with Tyrode’s buffer and lysed with 0.01% triton. The amount of bacteria in the epithelial cell lysate were determined by plating serial dilutions on LB agar. The experiment was performed in four replicates. The number of adhered bacteria were enumerated by subtracting mean of invaded bacteria (B) from mean of total host associated bacteria (A) and error (ΔZ) was calculated as (ΔZ)^2^ = (ΔA)^2^ + (ΔB)^2^ where, ΔA is SEM associated with the A and ΔB is SEM associated with B. The data were reported as log_10_ colony forming units (CFU) /well. Differences in the mean due to treatment were tested by one way ANOVA. Post ANOVA, the means were compared to each other by Tukey–Kramer’s method.

### Caspase assays

Caco2 cells were cultured in a 96-well plate as previously described and were washed with phosphate buffered saline (PBS) before infection with bacteria. Caco2 cells were infected with either *Bifidobacterium longum* spp. *infantis* ATCC 15697, *Salmonella sv.* Typhimurium LT2 ATCC 700720, or both organisms simultaneously. Upon adding bacterial treatments (MOI of 1:1000) in a volume of 50 μL, cells were incubated at 37°C with 5% CO_2_. After 8 h, caspase 8, 9 and 3/7 activities were measured using Caspase-Glo assay kits (Promega, Madison, WI) exactly as per manufacturer’s instructions. Bioluminescence due to caspase activity was measured using the DTX 880 Multimode Detector (Beckman Coulter, Brea, CA). Differences in the mean due to treatments were tested by one way ANOVA. Post ANOVA, the means were compared to each other by Tukey–Kramer’s method.

### Infection of epithelial cells for gene expression

Human colonic epithelial (Caco2) cells were cultured in T75 as described earlier, and were serum starved for 24 h prior to infection with bacteria. Caco2 cells were infected with either *Bifidobacterium longum* spp. *infantis* ATCC 15697, *Salmonella sv.* Typhimurium LT2 ATCC 700720, or both the organisms simultaneously at an MOI of 1:1000 in a final volume of 10 mL. All the three bacterial treatments were also incubated in absence of the Caco2 cells in the exact same conditions. All the infected cells were incubated at 37°C with 5% CO_2_. At 30, 60 and 120 min after infection, media with non-adherent bacteria was aspirated and 10 mL TRIzol LS reagent (Invitrogen, Carlsbad, CA) was added to the cells. This was gently mixed with pipette followed by centrifugation at 7200 × *g* for 5 min to pellet the bacteria. TRIzol LS supernatant was stored in a clean tube and further processed for RNA extraction from Caco2 cells. The bacterial pellet was re-suspended in 2 mL of fresh TRIzol LS, gently mixed and further processed for RNA extraction from host-associated bacteria.

### Bacterial RNA extraction and gene expression

The TRIzol LS suspension containing host-associated bacteria was centrifuged at 7200 × *g* for 5 min. The remaining TRIzol LS supernatant was stored in a clean tube for later use. 1 mL of lysis enzyme cocktail containing 50 mg/mL of lysozyme (Sigma) and 200 U/mL mutanolysin (Sigma) in TE buffer (10 mM Tris and 1 mM EDTA, pH 8) was added to bacterial pellet obtained by centrifugation. The solution was mixed gently and incubated at 37°C for 1 h followed by centrifugation at 7200 × *g* for 5 min. The supernatant was discarded, and the pellet was re-suspended in 250 μL of proteinase K buffer (100 mM Tris–HCl, 5 mM EDTA, 200 mM NaCl, and 0.2% SDS, pH 8) containing 8 U/mL of proteinase K (Fermentas, Glen Burnie, MD). This was incubated at 55°C for 1 h with intermittent mixing. To this, previously stored TRIzol LS was added and gently mixed. RNA was isolated from TRIzol LS exactly as per manufacturer’s recommendations. RNA concentration, A260/280 and A260/230 were measured on NanoDrop (Thermo scientific, Waltham, MA) and samples were processed further only if the RNA concentration was at least 0.5 μg/μl and ratios were ≥ 1.8. The RNA samples were analyzed for integrity on 2,100 Bioanalyzer (Agilent Technologies, Santa Clara, CA).

Total RNA (10 μg in 20 μL) was reverse transcribed into cDNA with random hexamers and Superscriptase II (Invitrogen, Carlsbad, CA) exactly as per manufacturer’s recommendations by using 400 U of Superscriptase II/10 μg RNA. Upon cDNA synthesis, the enzyme was heat inactivated and the RNA templates were degraded with 80 U of RNaseH (Epicentre, Madison, WI) at 37°C for 20 min. The reaction mixture was cleaned using the Qiaquick-PCR purification kit (Qiagen, Valencia, CA) exactly as per manufacturer’s instructions. Purified cDNA was eluted from the column twice with a total of 100 μL of nuclease free water (Ambion, Austin, TX).

cDNA was fragmented using DNaseI (Promega, Madison, WI) according to manufacturer’s recommendations by using 0.6 U of DNAseI/μg cDNA at 37°C for 20 min. The fragmented cDNA was labeled using GeneChip DNA Labeling reagent (Affymetrix, Santa Clara, CA) and Terminal Transferase enzyme (TdT) (New England Biolabs, Ipswich, MA) as per manufacturers’ recommendation by using 2 μL GeneChip labeling reagent and 3 μL TdT/μg cDNA at 37°C for 60 min. The samples were denatured prior to hybridization, at 98°C for 10 min followed by snap cooling at 4°C for 5 min.

Labeled cDNA was hybridized onto two different custom made Affymetrix GeneChip designed against all the annotated coding sequences of *Bifidobacterium longum* ssp. *infantis* ATCC 15697 (LoCascio et al., 2010) and *Salmonella enterica* subsp. *enterica sv.* Typhimurium LT2 ATCC 700720. Briefly the *Salmonella* array contained 9,852 probe sets, of which 4,735 probe sets were designed against *S. sv.* Typhimurium. Each probe set contained 11 probes, each 25 nucleotides long. The *Bifidobacteria* chip has been described by LoCascio et al. (2010). An aliquot (500 ng) of labeled cDNA for samples extracted from pure culture of *B. infantis* and *S.* Typhimurium; 1,000 ng labeled cDNA for samples extracted from co culture of *B. infantis* and *S.* Typhimurium; 2000 ng of labeled cDNA for samples extracted from co-culture of *B. infantis*/*S. sv.* Typhimurium and Caco-2; and 2,500 ng of labeled cDNA for samples extracted from co-culture of *B. infantis*, *S.* Typhimurium and Caco2 was hybridized onto the respective chips. In total, 24 chips *Salmonella* chips were processed (2 replicates X 3 time points (30 min, 60 min, 120 min) X 4 treatments) and 8 *Bifidobacteria* chips were processed (2 reps X 1 time point (120 min) X 4 treatments).

### Microarray data normalization and statistical analysis of microbial chips

Raw data (.cel files) was back ground corrected, quantile normalized and summarized using RMA-MS (Stevens et al., 2008). The resultant normalized log_2_ transformed intensity matrix was used for further statistical analysis. To detect differentially regulated genes, *Salmonella* chip data was analyzed as unpaired time course analysis with “signed area” as the time summary method, while for *Bifidobacteria* chips the data was analyzed as two class unpaired data, with T statistic, using Significance Analysis of Microarrays (SAM) (Tusher et al., 2001). All the genes were ranked based on the score (d) from SAM output. This pre-ordered ranked gene list was then used in Gene Set Enrichment Analysis software (GSEA) (Mootha et al., 2003; Subramanian et al., 2005) to detect the coordinate changes in the expression of groups of functionally related genes, upon respective treatments. Gene sets for *Bifidobacteria* were defined based on annotations from KEGG (Kanehisa et al., 2002), Cluster of Orthologous Groups of proteins (COGs) (Tatusov et al., 1997), Carbohydrate Active Enzyme Database CAZY (Park et al., 2010) and genes identified by LoCascio et al. (2010). Putative horizontally transferred genes in *B. infantis* were identified using Integrated microbial genomes system (IMG) (Markowitz et al., 2010). Genes sets based on protein localization were created based on annotations from CoBaltDB (Goudenège et al., 2010). Gene sets for *Salmonella* were defined based the annotations from Comprehensive Microbial Resource (CMR) (Peterson et al., 2001), Cluster of Orthologous Groups of proteins (COGs) (Tatusov et al., 1997), Virulence Factors of pathogenic bacteria DataBase (VFDB) (Chen et al., 2003; Yang et al., 2008), Carbohydrate Active Enzyme Database (CAZY) (Park et al., 2010), and BioCyc (Caspi et al., 2008). Gene sets based on putative horizontally transferred genes in *Salmonella* were defined using predictions from the Horizontal Gene Transfer Database (HGT-DB) (Garcia-Vallve et al., 2003). Genes sets based on protein localization were created using predictions from CoBaltDB (Goudenège et al., 2010).

### Caco-2 RNA extraction and gene expression

The TRIzol LS (Thermo Fisher Scientific, Waltham, MA, USA) supernatant obtained after pelleting the bacteria was freeze thawed twice in liquid nitrogen. 250 μL water was added to 750 μL of TRIzol LS sample. This was further processed for RNA extraction exactly as per manufacturer’s instructions. RNA concentration, A260/280 and A260/230 were measured on NanoDrop (Thermo scientific, Waltham, MA) and samples were processed further only if the RNA concentration was at least 0.5 μg/μl and ratios were ≥ 1.8. The RNA samples were analyzed for integrity on 2,100 Bioanalyzer (Agilent Technologies, Santa Clara, CA). Synthesis of cDNA, biotin labeled cRNA, fragmentation and purification of cRNA were carried out using one-cycle cDNA synthesis kit (Affymetrix, Santa Clara, CA) exactly as per manufacturer’s instructions. 10 μg of labeled and fragmented cRNA was hybridized onto the Affymetrix HGU133Plus2 GeneChips as per manufacturer’s recommendations at the Center for Integrated BioSystems (Utah State University, Logan, UT).

### Microarray data normalization and statistical analysis of the HGU133 Plus2 chips

Raw data (.cel files) were background corrected, quantile normalized and summarized using RMA (Irizarry et al., 2003). RMA normalized data was then filtered through the PANP algorithm (Warren et al., 2007) to make presence-absence calls for each probe set. Probe sets that were called present in at least one of the samples were included in further statistical analysis while rests were excluded. The resultant normalized, filtered, log_2_ transformed intensity matrix was analyzed as two class unpaired time course data with “signed-area” as the time summary method, using Significance Analysis of Microarrays (SAM) (Tusher et al., 2001). All the genes were ranked based on the score (d) obtained from SAM. This pre ordered ranked gene list was used as an input for Gene Set Enrichment Analysis software (GSEA) (Subramanian et al., 2005) to detect the coordinate changes in the expression of groups of functionally related genes, upon respective treatments. The gene sets were based on Gene Ontology annotations, KEGG annotations, Biocarta annotations and TRANSFAC annotations and were downloaded from the molecular signatures database (Subramanian et al., 2005). Ingenuity pathway analysis (IPA) was used to map expression data onto canonical pathways. Hierarchical clustering with multiscale bootstrapping (1,000 times) of all the samples based on normalized data was performed using PVCLUST (Suzuki and Shimodaira, 2006).

### Western blots

Relative abundance of target proteins was quantified by densitometric analysis of western blots. Caco2 cells were cultured and infected exactly as with those infected for the gene expression, detailed above. Cells from the T75 were removed from the flask by scraping; suspended in 1 mL protease/phosphatase inhibitor cocktail (30 mM HEPES, 1 mM EDTA, 50 mM Sodium pyrophosphate, 100 mM sodium fluoride, 10 mM orthovanadate, 1 Roche protease inhibitor cocktail tablet/50 mL solvent) and lysed using a bead beater at full speed with 3 pulses of 30 s each with intermittent incubation on ice for 1 min. The cell lysates were stored at −70°C for further analysis. 50 μg protein was diluted in 1X SDS sample buffer and the samples were heated at 95°C for 10 min and centrifuged at 12,000 X g for 10 min. The sample was resolved on precast 10% Tris–HCl polyacrylimide gels (Bio-rad Laboratories, Hercules, CA) at a constant current of 45 mAmp per gel using the Criterion electrophoresis system (Bio-rad Laboratories, Hercules, CA). The resolved proteins were then transferred to a PVDF membrane (Bio-rad Laboratories, Hercules, CA) using the Trans-Blot semi-dry electrophorectic cell (Bio-rad Laboratories, Hercules, CA) as per the manufacturer’s recommendation. Upon completion of the transfer, the blots were probed for the presence of p-Akt 473 (#9271) and p-AKT 308 (#9275) by Pierce® Fast Western Blot Kit (Thermo Fisher Scientific, IL, USA), according to the manufacturer’s recommendations. Primary antibodies for all the proteins were purchased from Cell Signaling Tech. (Boston, MA). The blots were imaged using a Kodak Image Station 2000R (Carestream Health, Rochester, NY). Densitometric analysis of the blot was done using Image J (Abramoff et al., 2004). Differences in the means due to treatment were tested by two-way ANOVA with repeated measures.

### Small metabolite profiling

Extracellular supernatant and intracellular cell lysate (50 μL) collected from the infection experiments were extracted with an equal volume of ice-cold methanol and incubated at −20°C for 60 min. The resultant precipitates were separated from the sample by centrifugation at 20,000 X g for 10 min. The supernatant was evaporated to dry using a speed-vac at room temperature. Metabolite profiles for all the samples were determined using GC–MS at the UC Davis Genome Center (Davis, CA) as described previously (Fiehn et al., 2008). In brief, the dried sample was derivatized using N-methyl-N-(trimethylsilyl)-trifluoroacetamide (Sigma, St. Louis, MO) and spiked with retention index markers (RI) as described previously (Fiehn et al., 2008). The samples were analyzed on Agilent 6,890 gas chromatograph controlled using Leco ChromaTOF software version 2.32. as described previously (Fiehn et al., 2008). GC/MS peaks were annotated based on the mass spectra and retention index using the BinBase database (Fiehn et al., 2005). Peak areas were normalized by calculating the sum area of all identified compounds for each and subsequently dividing all data associated with a sample by the corresponding metabolite sum. The resulting data were multiplied by a constant factor in order to obtain values without decimal places and, the peak intensities were log_2_ transformed to remove the heteroscedasticity (Fiehn et al., 2008). Hierarchical clustering with multiscale bootstrapping (1,000 times) of all the samples based on normalized data was performed using PVCLUST (Suzuki and Shimodaira, 2006).

## Data availability statement

Expression data can be found in the GEO repository under accession number GSE266880.

## Ethics statement

Ethical approval was not required for the studies on humans in accordance with the local legislation and institutional requirements because only commercially available established cell lines were used.

## Author contributions

CS: Data curation, Formal analysis, Visualization, Writing – original draft, Writing – review & editing. BW: Conceptualization, Formal analysis, Funding acquisition, Investigation, Methodology, Project administration, Resources, Software, Supervision, Validation, Writing – original draft, Writing – review & editing. RG: Conceptualization, Data curation, Formal analysis, Investigation, Methodology, Validation, Visualization, Writing – original draft, Writing – review & editing. PD: Conceptualization, Data curation, Formal analysis, Investigation, Methodology, Software, Validation, Visualization, Writing – original draft, Writing – review & editing. JS: Conceptualization, Data curation, Formal analysis, Investigation, Methodology, Software, Validation, Visualization, Writing – original draft, Writing – review & editing.
