# Supplementary figures and images for: The Yin and Yang of pathogens and probiotics: interplay between Salmonella enterica sv. Typhimurium and Bifidobacterium infantis during co-infection
Source: Front Microbiol. 2024 May 15;15:1387498. doi: 10.3389/fmicb.2024.1387498 (PMC11133690; doi:10.3389/fmicb.2024.1387498)

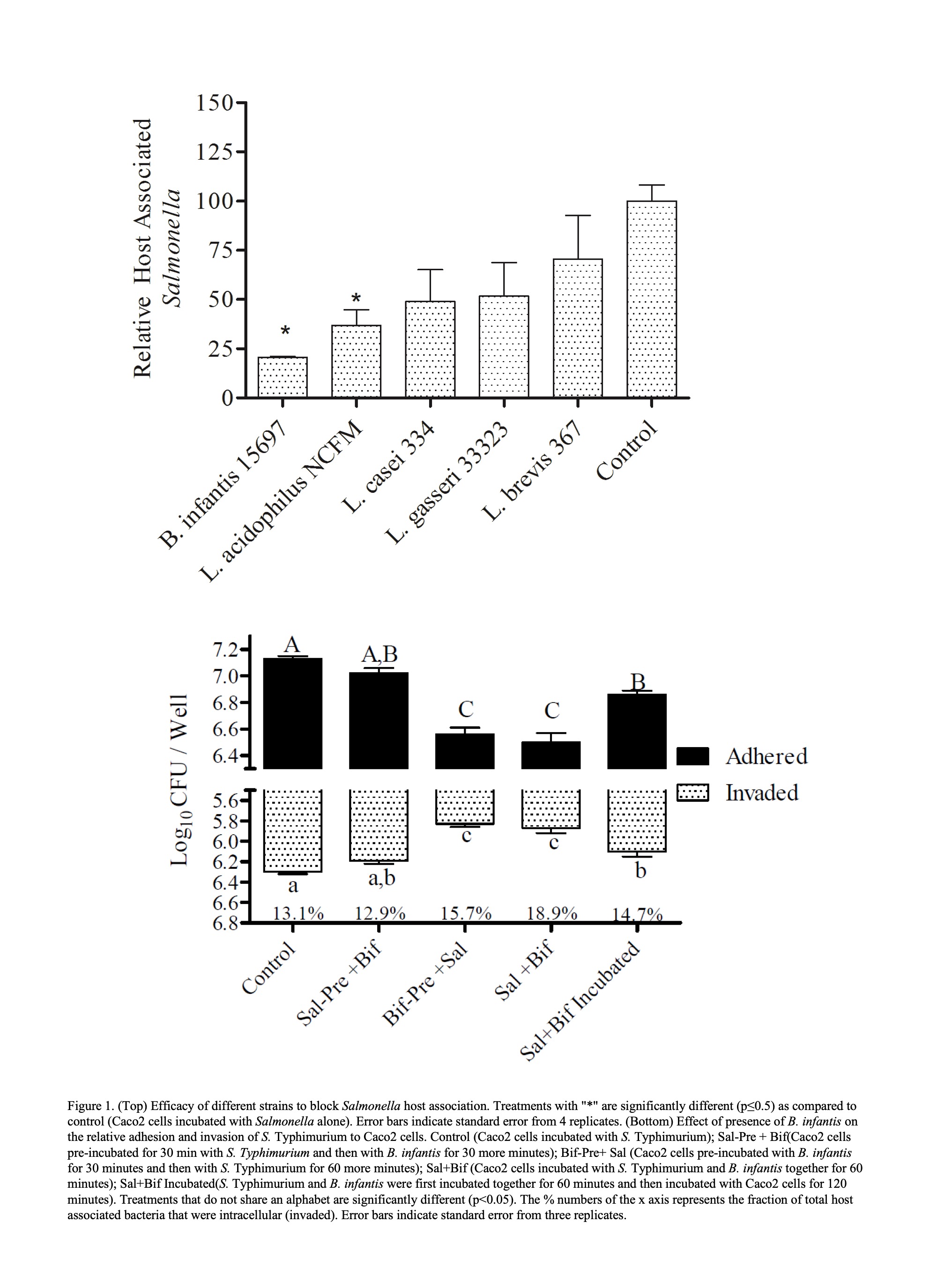

Supplement: Supplementary file 1 [file Figure_1.JPEG]

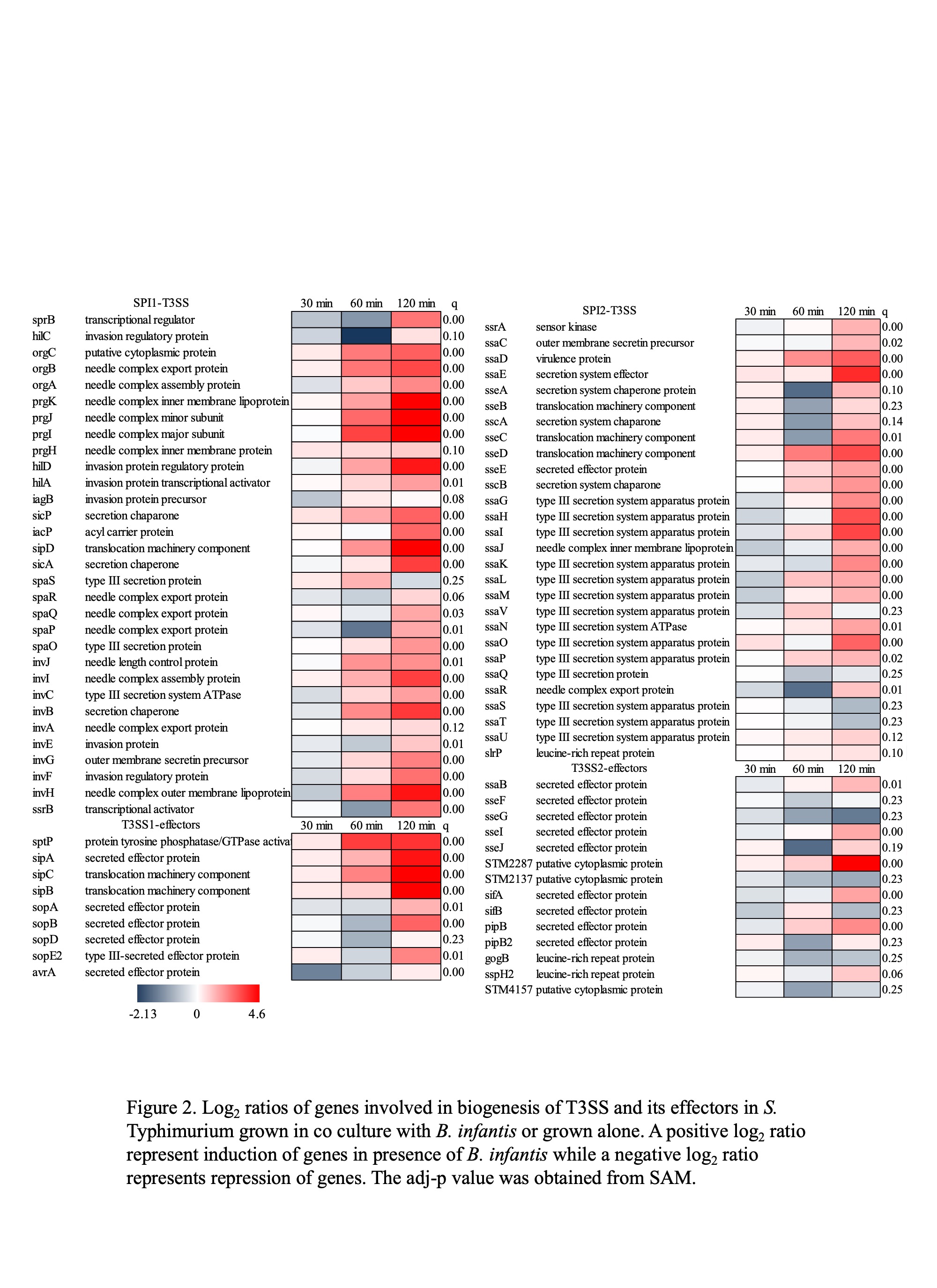

Supplement: Supplementary file 2 [file Figure_2.JPEG]

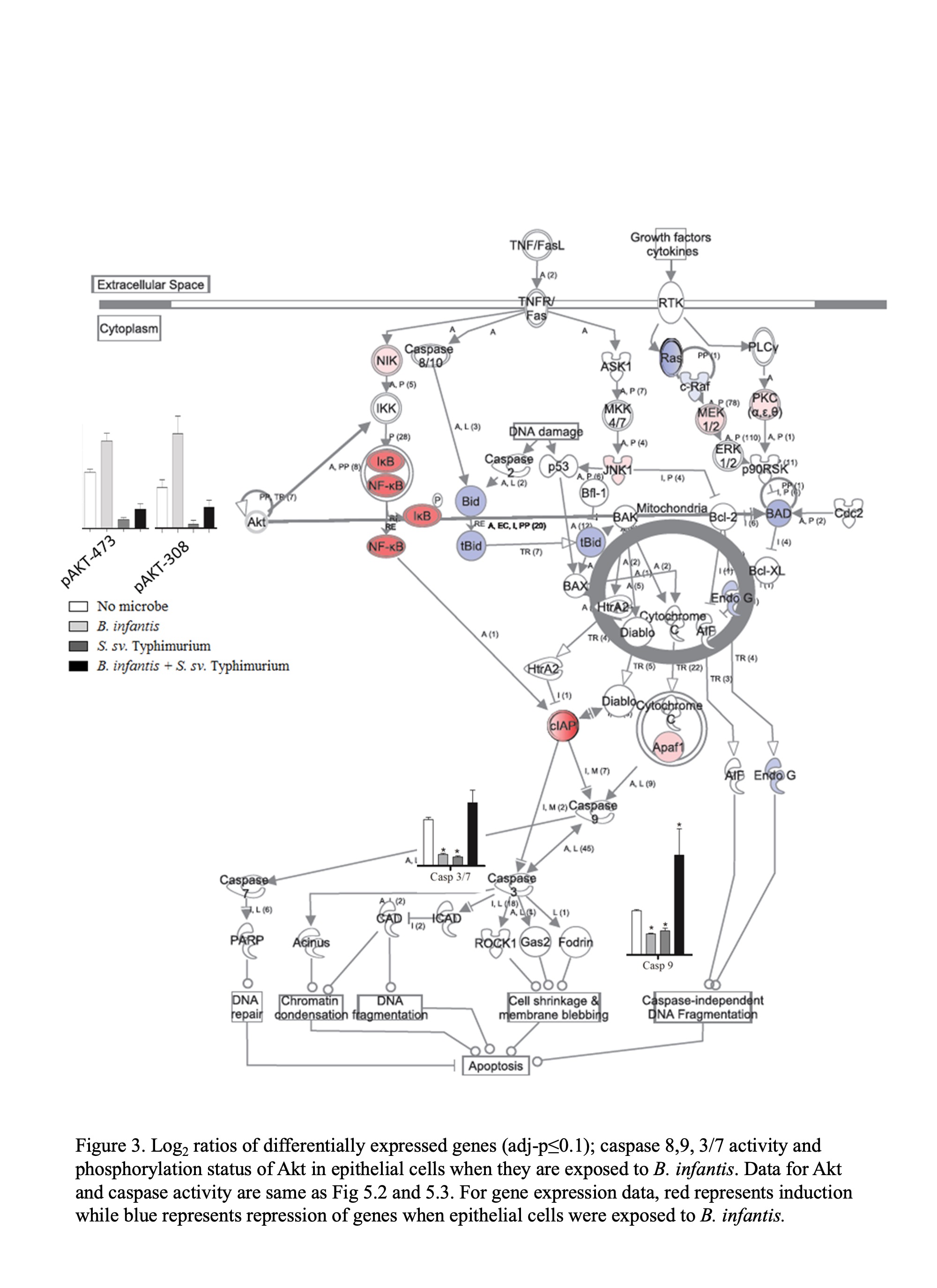

Supplement: Supplementary file 3 [file Figure_3.JPEG]

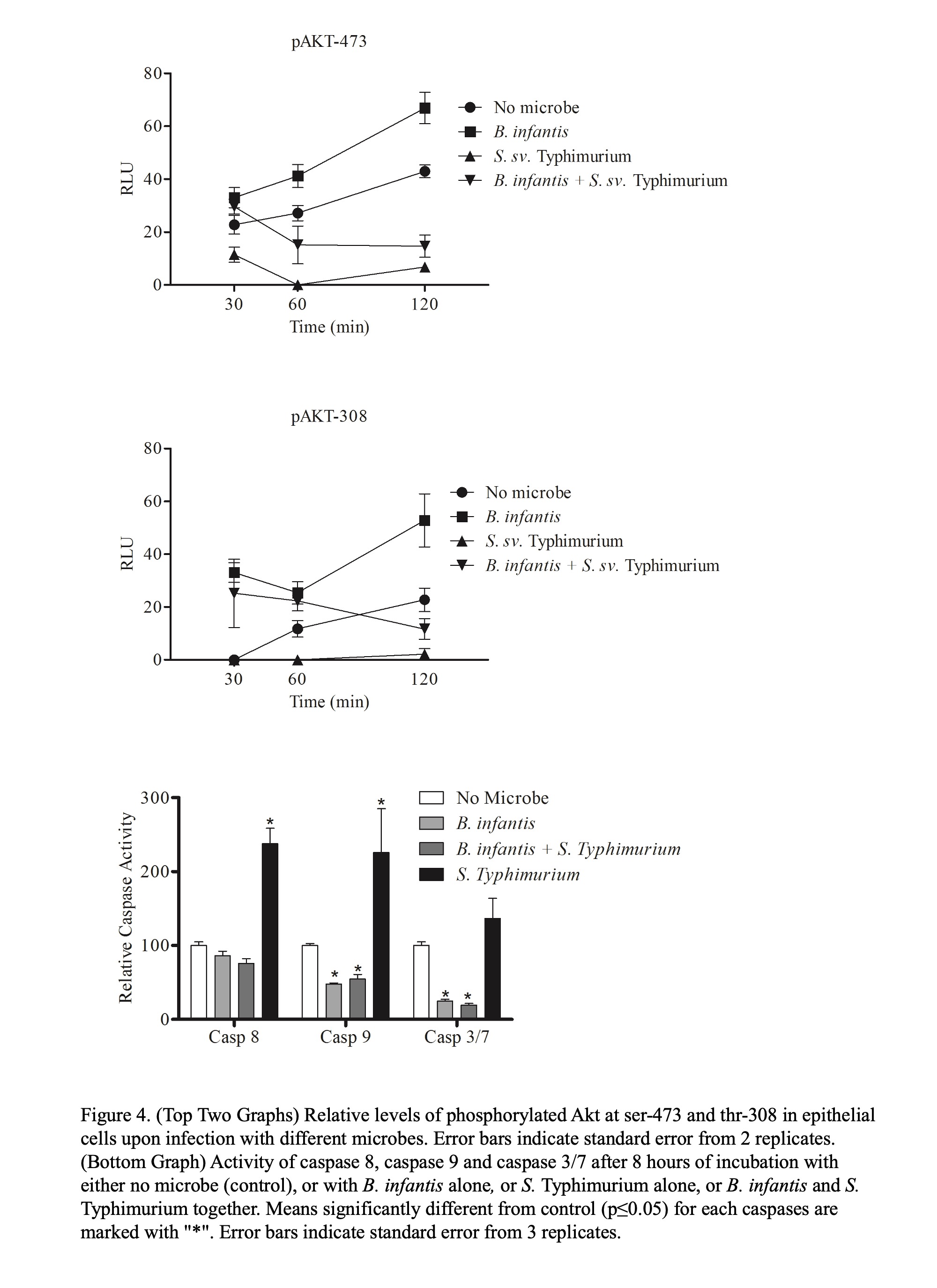

Supplement: Supplementary file 4 [file Figure_4.JPEG]

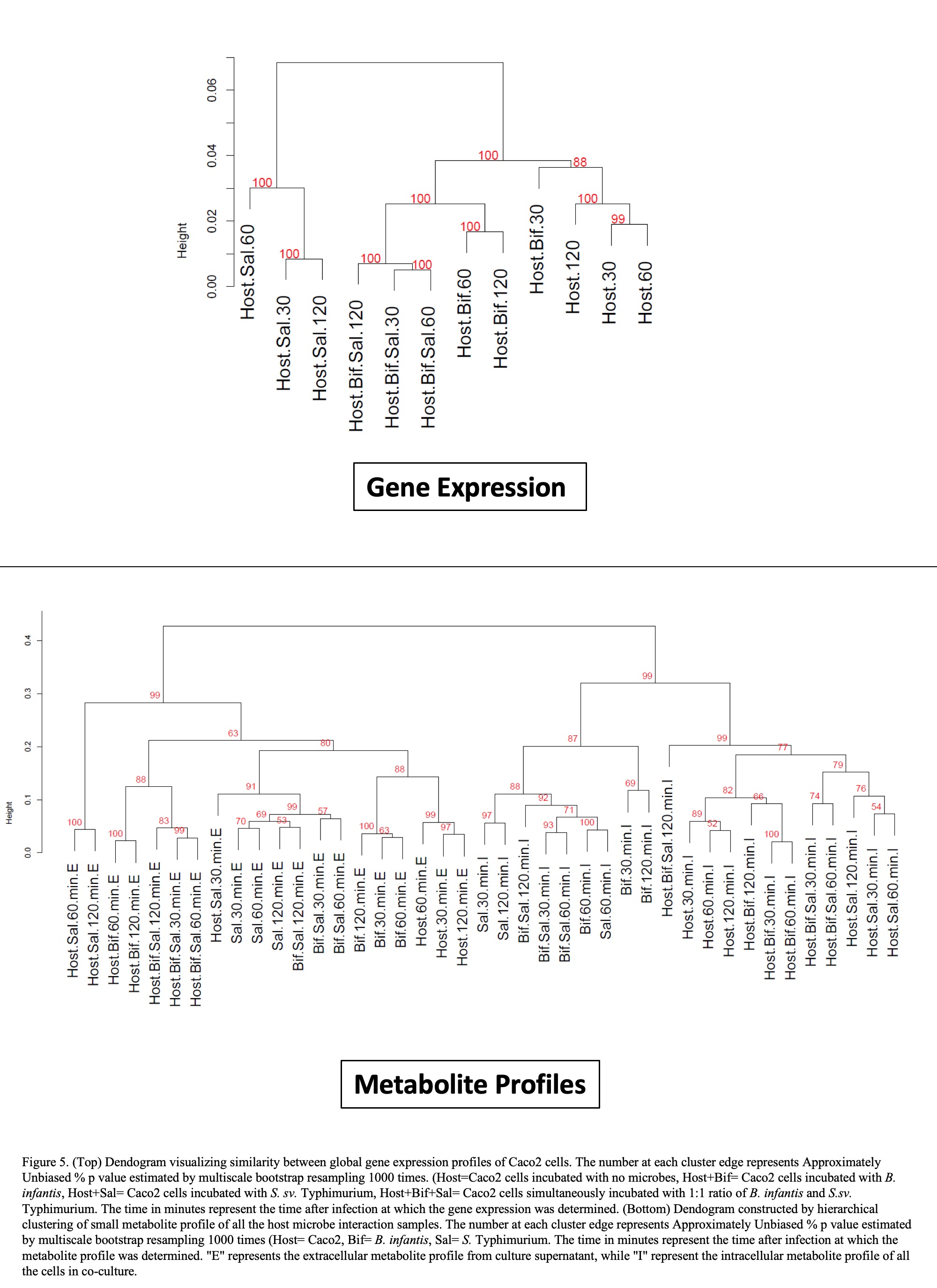

Supplement: Supplementary file 5 [file Figure_5.JPEG]

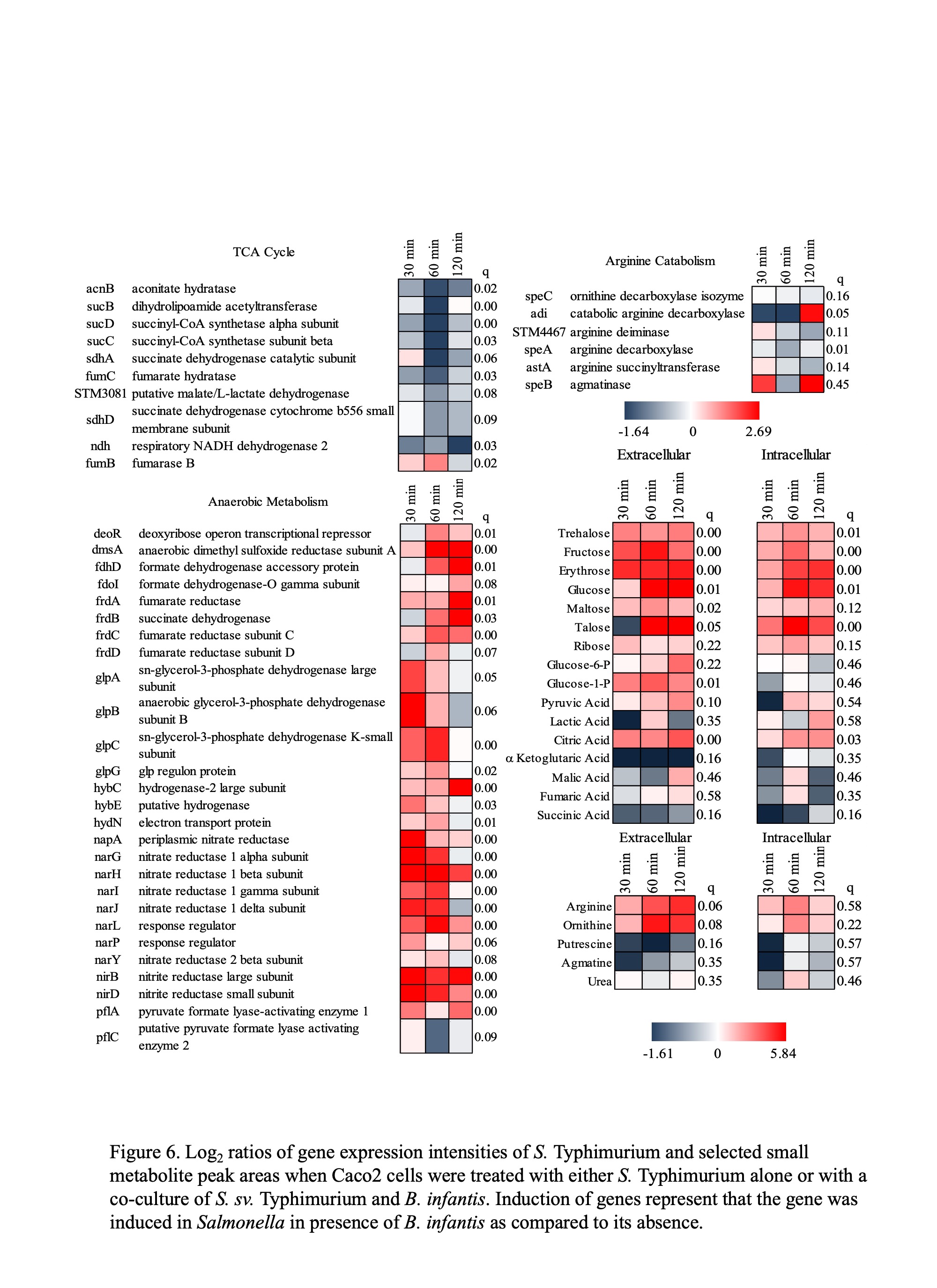

Supplement: Supplementary file 6 [file Figure_6.JPEG]
