# Supplementary material for: The Yin and Yang of pathogens and probiotics: interplay between Salmonella enterica sv. Typhimurium and Bifidobacterium infantis during co-infection
Source: Front Microbiol. 2024 May 15;15:1387498. doi: 10.3389/fmicb.2024.1387498 (PMC11133690; doi:10.3389/fmicb.2024.1387498)
Supplement: Supplementary file 7 [file Table_1.DOCX]

Table S1 Differentially regulated pathways (q≤0.05) in *S.* Typhimurium when exposed to *B. infantis* as compared to normal growth. NES= Normalized enrichment score. A positive score indicates that the gene set was induced when exposed to *B. infantis*, while a negative score indicated that the gene set was repressed.

| **Gene Set** | Size of Gene Set | Genes Regulated | NES | Adj-P Val |
| --- | --- | --- | --- | --- |
| Amino Acid Biosynthesis- Glutamate Family | 19 | 8 | 1.57 | 0.05 |
| Amino Acid Biosynthesis- Serine Family | 21 | 5 | 1.55 | 0.05 |
| Biosynthesis Of Cofactors- Prosthetic Groups- And Carriers- Glutathione And Analogs | 6 | 5 | 1.55 | 0.05 |
| Cell Envelope- Biosynthesis And Degradation Of Surface Polysaccharides And Lipopolysaccharides | 82 | 29 | 1.86 | 0.00 |
| Cellular Processes- Chemotaxis And Motility | 40 | 22 | 1.68 | 0.02 |
| Cellular Processes- Detoxification | 28 | 16 | 1.56 | 0.05 |
| Cog F-Nucleotide Transport And Metabolism | 80 | 56 | 1.87 | 0.00 |
| Cog J-Translation | 170 | 94 | 2.27 | 0.00 |
| Cog M-Cell Wall Membrane Biogenesis | 237 | 85 | 1.70 | 0.01 |
| Cog O-Posttranslational Modification Protein Turnover Chaperones | 142 | 70 | 1.57 | 0.05 |
| Energy Metabolism- ATP-Proton Motive Force Interconversion | 15 | 7 | 1.62 | 0.03 |
| Energy Metabolism- Pyruvate Dehydrogenase | 8 | 6 | 1.73 | 0.01 |
| Fatty Acid And Phospholipid Metabolism- Biosynthesis | 44 | 24 | 1.55 | 0.05 |
| Fur Regulon | 66 | 33 | 1.87 | 0.00 |
| Genes Induced By Qse Two Component System | 44 | 15 | 1.65 | 0.02 |
| Genes Regulated By HilA | 17 | 11 | 1.68 | 0.02 |
| Genes Regulated By SsrA-SsrB Two Component System | 117 | 55 | 1.55 | 0.05 |
| Glycan Biosynthesis-Lps Biosynthesis | 27 | 23 | 1.85 | 0.00 |
| Membrane Transport-Pores Ion Channels | 35 | 15 | 1.86 | 0.00 |
| Membrane Transport-Protein Export | 16 | 14 | 1.64 | 0.02 |
| Membrane Transport-Secretion System | 43 | 27 | 1.66 | 0.02 |
| Protein Synthesis- Translation Factors | 28 | 19 | 1.80 | 0.00 |
| Purines- Pyrimidines- Nucleosides- And Nucleotides- 2-Deoxyribonucleotide Metabolism | 10 | 6 | 1.70 | 0.02 |
| Purines- Pyrimidines- Nucleosides- And Nucleotides- Purine Ribonucleotide Biosynthesis | 18 | 16 | 1.77 | 0.01 |
| SPI-1 | 40 | 25 | 2.04 | 0.00 |
| SPI1-T3SS | 30 | 21 | 1.79 | 0.00 |
| SPI2-T3SS | 27 | 18 | 1.58 | 0.04 |
| T3SS | 58 | 42 | 1.90 | 0.00 |
| T3SS Effectors | 22 | 12 | 1.56 | 0.05 |
| Adherence Factors-Fimbrial | 66 | 29 | -2.61 | 0.00 |
| Cellular Processes- DNA Transformation | 30 | 29 | -3.82 | 0.00 |
| Genes With % GC Greater Than 60 | 196 | 102 | -2.04 | 0.00 |
| Membrane Transport-Electron Transfer Carriers | 31 | 20 | -2.20 | 0.00 |
| Mobile And Extra chromosomal Element Functions- Plasmid Functions | 28 | 26 | -3.68 | 0.00 |
| SPV Locus | 4 | 3 | -1.87 | 0.02 |
