# Supplementary material for: The Yin and Yang of pathogens and probiotics: interplay between Salmonella enterica sv. Typhimurium and Bifidobacterium infantis during co-infection
Source: Front Microbiol. 2024 May 15;15:1387498. doi: 10.3389/fmicb.2024.1387498 (PMC11133690; doi:10.3389/fmicb.2024.1387498)
Supplement: Supplementary file 8 [file Table_2.DOCX]

Table S2 Differentially regulated pathways (q≤0.20) in Caco2 cells when exposed to *B. infantis* and *S.*Typhimurium as compared to when exposed to only *B. infantis*. NES= Normalized enrichment score. A positive score indicates that the gene set was induced in Caco2 cells when exposed to *B. infantis* and *S.*Typhimurium, while a negative score indicated that the gene set was repressed.

| **Gene Set** | Size of Gene Set | Genes Regulated | NES | Adj-p Val |
| --- | --- | --- | --- | --- |
| **Kegg Pathway** | | | | |
| Nod Like Receptor Signaling Pathway | 33 | 7 | -2.17 | 0.01 |
| Steroid Hormone Biosynthesis | 23 | 3 | -2.10 | 0.01 |
| **Biocarta Pathway** | | | | |
| Ataxia Telangiectasia-mutated gene Pathway | 18 | 10 | -2.12 | 0.01 |
| Il6 Pathway | 19 | 9 | -2.00 | 0.02 |
| **GO Annotations** | | | | |
| Cell Junction | 48 | 22 | -2 | 0.13 |
| Regulation Of Cell Migration | 14 | 8 | -1.97 | 0.13 |
| Basolateral Plasma Membrane | 22 | 9 | -1.93 | 0.14 |
| Chemokine Receptor Binding | 10 | 4 | -1.94 | 0.15 |
| Chemokine Activity | 10 | 4 | -1.95 | 0.16 |
| Cell Matrix Junction | 10 | 6 | -2.02 | 0.20 |
