# Supplementary material for: The Yin and Yang of pathogens and probiotics: interplay between Salmonella enterica sv. Typhimurium and Bifidobacterium infantis during co-infection
Source: Front Microbiol. 2024 May 15;15:1387498. doi: 10.3389/fmicb.2024.1387498 (PMC11133690; doi:10.3389/fmicb.2024.1387498)
Supplement: Supplementary file 9 [file Table_3.DOCX]

Table S3 Transcriptional regulons that are differentially regulated (adj-p≤0) in Caco-2 cells when they were exposed to *B. infantis* and *S.*Typhimurium as compared to when they were exposed to only *B. infantis*. NES= Normalized enrichment score. A positive score indicates that the gene set was induced in Caco-2 cells when exposed to *B. infantis* and *S.* Typhimurium, while a negative score indicated that the gene set was repressed.

| Gene Set | Binding Motif | Transcription Factor | Remarks | Size | Genes Regulated | NES | Adj-p Val |
| --- | --- | --- | --- | --- | --- | --- | --- |
| V$CREB_Q2 | NSTGACGTAANN | CREB1: cAMP responsive element binding protein 1 | Activated by a variety of stimuli leading to phosphorylation of CREB1 mediated by PKA principally through GPCR signaling | 140 | 29 | -2.17 | 0 |
| V$CREB_Q4_01 | CNNTGACGTMA | CREB1: cAMP responsive element binding protein 1 |  | 129 | 40 | -2.25 | 0 |
| V$CREB_Q4 | NSTGACGTMANN | CREB1: cAMP responsive element binding protein 1 |  | 109 | 25 | -1.99 | 0 |
| V$CREBP1_Q2 | VGTGACGTMACN | ATF2: activating transcription factor 2 | This protein binds to the cAMP-responsive element (CRE),The protein forms a homodimer or heterodimer with c-Jun and stimulates CRE-dependent transcription. | 134 | 31 | -1.99 | 0 |
