# Supplementary material for: The Yin and Yang of pathogens and probiotics: interplay between Salmonella enterica sv. Typhimurium and Bifidobacterium infantis during co-infection
Source: Front Microbiol. 2024 May 15;15:1387498. doi: 10.3389/fmicb.2024.1387498 (PMC11133690; doi:10.3389/fmicb.2024.1387498)
Supplement: Supplementary file 11 [file Table_5.DOCX]

Table S5 Differentially regulated genesets in *B. infantis* after 120 minutes of incubation with *S.* Typhimurium as compared to *B. infantis* incubated alone. NES= Normalized enrichment score. A positive score indicates that the gene set was induced in *B. infantis* when exposed to *S..* Typhimurium, while a negative score indicated that the gene set was repressed

| **Gene Set** | Size of Gene Set | Genes Regulated | NES | Adj-p Val |
| --- | --- | --- | --- | --- |
| Genes with %GC >70 | 48 | 24 | 2.64 | 0.00 |
| Cog J Translation | 137 | 66 | 1.97 | 0.01 |
| Sec System | 9 | 7 | 1.96 | 0.01 |
| Translation | 57 | 28 | 1.78 | 0.04 |
| Other Transcription Related Proteins | 4 | 4 | 1.75 | 0.05 |
| Purine Metabolism | 43 | 17 | 1.66 | 0.09 |
| Drug Metabolism | 5 | 4 | 1.65 | 0.08 |
| Histidine Metabolism | 9 | 8 | 1.62 | 0.10 |
| Multi Transmembrane | 460 | 198 | -1.91 | 0.02 |
| Cog G Carbohydrate Transport and Metabolism | 191 | 84 | -1.90 | 0.01 |
| Glycosyl Hydrolases | 41 | 17 | -1.86 | 0.02 |
| Cog P-Inorganic Ion Transport and Metabolism | 77 | 19 | -1.78 | 0.03 |
| Sec Pi Substrate | 574 | 235 | -1.70 | 0.06 |
| Cog V Defense Mechanism | 69 | 39 | -1.68 | 0.06 |
