# Supplementary material for: The Yin and Yang of pathogens and probiotics: interplay between Salmonella enterica sv. Typhimurium and Bifidobacterium infantis during co-infection
Source: Front Microbiol. 2024 May 15;15:1387498. doi: 10.3389/fmicb.2024.1387498 (PMC11133690; doi:10.3389/fmicb.2024.1387498)
Supplement: Supplementary file 12 [file Table_6.docx]

Table S6 Gene sets that are conserved in *B. infantis* but divergent in *B. longum* based on comparative genomic hybridization of 15 *Bifidobacteria* strains (adj-p≤0.07).

| **Gene Set** | Size of Gene Set | Divergent Genes | NES | Adj-p  Val |
| --- | --- | --- | --- | --- |
| ABC Transporters | 76 | 50 | -1.93 | 0.00 |
| Cog G Carbohydrate Transport and Metabolism | 191 | 82 | -1.68 | 0.01 |
| Cog V Defense Mechanism | 69 | 35 | -1.64 | 0.01 |
| Genes %GC Less Than 40 | 34 | 32 | -1.79 | 0.00 |
| Genes with %GC Between 40 And 50 | 108 | 81 | -1.82 | 0.00 |
| HGT from Actinomycetales | 109 | 50 | -1.81 | 0.00 |
| HGT from Firmicutes | 114 | 81 | -1.98 | 0.00 |
| Human Milk Oligosaccharide Utilization Cluster | 61 | 50 | -2.32 | 0.00 |
| Lipid Anchored | 61 | 31 | -1.58 | 0.03 |
| Multi Transmembrane | 460 | 161 | -1.5 | 0.07 |
| Protein Folding and Associated Processing | 13 | 4 | -1.63 | 0.01 |
| Sec PII Substrate | 69 | 34 | -1.61 | 0.02 |
